# Involvement of Serotonergic and Dopaminergic Systems in *Aloysia gratissima* var. *gratissima*: Antidepressant-like Effect, UPLC-DAD-MS Chemical Characterization, and Computational Evidence

**DOI:** 10.3390/ph19020329

**Published:** 2026-02-17

**Authors:** Miguel A. Campuzano-Bublitz, Alberto Burgos-Edwards, Elvio Gayozo, Adelian A. Acosta, Rodrigo S. Paredes, Alex D. Campuzano-Kennedy, Antonia K. Galeano, Yenny P. González, Nelson L. Alvarenga, Teresa Taboada-Jara, María L. Kennedy

**Affiliations:** 1 Departamento de Farmacología, Facultad de Ciencias Químicas, Universidad Nacional de Asunción, Campus Una, San Lorenzo 2169, Paraguay; mbublitz@qui.una.py (M.A.C.-B.); alejandradasilvasoares@gmail.com (A.A.A.); rodbusiness365@gmail.com (R.S.P.); alexcamp.kenn@gmail.com (A.D.C.-K.); agaleano@qui.una.py (A.K.G.); ttaboada@qui.una.py (T.T.-J.); 2 Departamento de Fitoquímica, Facultad de Ciencias Químicas, Universidad Nacional de Asunción, Campus Una, San Lorenzo 2169, Paraguay; aburgos@qui.una.py (A.B.-E.); nelson@qui.una.py (N.L.A.); 3 Departamento de Biología, Facultad de Ciencias Exactas y Naturales, Universidad Nacional de Asunción, Campus Una, San Lorenzo 2169, Paraguay; elviologo@gmail.com; 4 Departamento de Botánica, Facultad de Ciencias Químicas, Universidad Nacional de Asunción, Campus Una, San Lorenzo 2169, Paraguay; ygonzale@qui.una.py

**Keywords:** tail suspension test, forced swimming test, WAY100635, ketanserin, ondansetron, haloperidol, prazosin, bicuculline

## Abstract

**Background/Objectives:** As the prevalence of depression and the use of antidepressants have risen steadily in the last decade, new treatment options are needed. *Aloysia gratissima* var. *gratissima* ethanol extract has previously shown antidepressant-like activity, and the present study was conducted to identify the active fraction and clarify the possible mechanisms of action. **Methods:** Tail suspension (TST) and forced swimming (FST) behavioral tests were performed, and possible mechanisms of action were elucidated using serotonergic, dopaminergic, adrenergic, and GABAergic system antagonists. UPLC-DAD-MS analyses were performed to identify compounds in active fractions, and molecular docking studies were carried out to determine the binding affinities of these compounds to serotonergic and dopaminergic receptors (5-HT_1A_, 5-HT_2A_, 5-HT_3_, and D_2_R). **Results:** Ethyl acetate and butanol fractions were found to decrease immobility time in FST. The reduction in immobility time during the FST caused by the ethyl acetate fraction was reversed by pretreating mice with WAY100635 (5-HT_1A_ antagonist), ketanserin (a 5-HT_2A_ antagonist, ondansetron (5-HT_3_ antagonist), or haloperidol (D_2_ antagonist). UPLC-DAD-MS analysis revealed a similar composition for the ethyl acetate and butanol fractions of *A. gratissima* var. *gratissima*. Pharmacokinetic predictions suggest that only a few of the identified compounds have the potential to permeate the blood–brain barrier, and molecular docking simulations showed that compounds such as 13-oxooctadecadienoic acid, ferulic acid, and coumaric acid have binding affinities to the druggable site of serotonergic and dopaminergic receptors. **Conclusions:** These results suggest that the Agg ethyl acetate fraction possesses antidepressant-like activities, altering dopaminergic and serotonergic system functions. Computational simulations also suggest that some of the identified compounds have binding affinities to the 5-HT_1A_, 5-HT_2A_, 5-HT_3_, and D_2_R receptors.

## 1. Introduction

Around 4% of the world’s population suffers from depression, one of the most common mental disorders [1]. Access to treatment is insufficient, considering that only about one-third of people with depression receive mental health treatment in developed countries [2]. Furthermore, the delayed improvement in mood (at least two weeks) and the undesirable side effects that accompany treatment contribute to patients tending to discontinue or prematurely discontinue their medication [3]. Therefore, despite the number of antidepressants currently available, further advances are needed from a clinical perspective [4].

The pathophysiology of depression is multifactorial and has not yet been fully defined. It includes monoamine imbalances such as serotonin (5-HT), noradrenaline (NA), and/or dopamine (DA) [5], in addition to other mechanisms such as hypothalamic–pituitary–adrenal axis dysfunction, altered neuroplasticity, reduced neurotrophic factor in the brain, inflammation, and gut microbiome imbalance [4]. Furthermore, new treatment options could include gamma-aminobutyric acid (GABA) type A receptor modulators and microbiota manipulation [4,5,6,7,8].

First-line therapy for depression includes selective serotonin reuptake inhibitors, serotonin–norepinephrine reuptake inhibitors, serotonin modulators, and atypical antidepressants. Current challenges in antidepressant treatment include long-term safety, use during pregnancy, dosage and cost reduction, and the risk of side effects and adverse effects [9].

Herbal medicine and traditional medicine are accessible alternative treatments that have long been used to treat all kinds of illnesses, including depression [6], primarily in developing countries and rural communities [7]. Various medicinal plants have demonstrated efficacy in treating central nervous system disorders, with the advantages of a relatively low cost and their status as the primary form of therapy in some communities in developing countries. This is due to limited or poor access to allopathic medicine [8]. Natural compounds such as curcumin [10], α-mangostin [11], and myrsinoic acid [12] have been identified as potential new antidepressants or as alternatives to enhance the current treatment of depression. Curcumin possibly interacts with the serotonergic system and the GABA_A_ receptor; α-mangostin modifies the GABAergic, serotonergic, and dopaminergic systems; and myrsinoic acid can normalize depressive-like behaviors, influencing the monoaminergic system and inhibiting Monoamine Oxidase A and pathways related to inflammation and oxidative stress [10,11,12].

We previously investigated the antidepressant-like effects of *Aloysia gratissima* var. *gratissima* (Verbenaceae) in mice. The ethanol extract of the aerial parts, orally administered for 7 days, reduced the immobility time in the forced swimming test by approximately 77% compared to a control group [13]. In this study, we report on the bio-guided fractionation of *A. gratissima* var. *gratissima* into hexane, dichloromethane, ethyl acetate, and butanol fractions. Once the active fraction was identified, the potential mechanism of action was analyzed. For this purpose, serotonin, dopamine, norepinephrine, and GABA_A_ receptor antagonists were used. Additionally, the major constituents from active fractions were tentatively identified, and their binding affinities to receptors involved in serotonergic and dopaminergic pathways (5-HT_1A_, 5-HT_2A_, 5-HT_3_, and D_2_R) were analyzed employing computational methods, including molecular docking simulations, pharmacokinetics, and oral bioavailability predictions.

## 2. Results

### 2.1. Assessment of Oral Treatment Effect of A. gratissima var. gratissima Fractions in the Tail Suspension Test and the Forced Swimming Test

We extracted hexane, dichloromethane, ethyl acetate, and butanol fractions from *Aloysia gratissima* var. *gratissima* (Agg) ethanol extract. Agg powder (151 g) was extracted with ethanol, yielding 25.7 g (17%). From 10 g of the extract, 0.5 g (5%) was dissolved in hexane, 2.1 g (21%) in dichloromethane, 0.31 g (3.1%) in ethyl acetate, and 0.63 g (6.3%) in butanol. To identify the active fraction, the animals were treated with 0.1 and 1 mg/kg of each fraction, water (vehicle; control), or the reference drug imipramine, with each receiving three treatments over 24 h, i.e., at 24, 18, and 1 h before being subjected to the behavior test. In other groups, treatment was administered once daily for 7 consecutive days. One hour after the treatment, the animals were first subjected to the tail suspension test (TST) and, subsequently, to the forced swimming test (FST).

**Figure 1:**
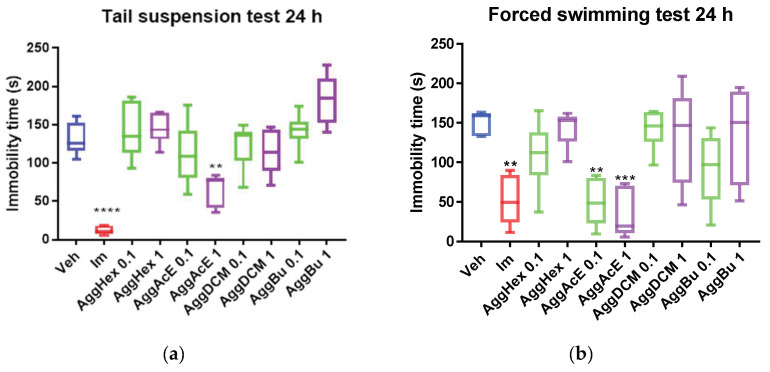
Immobility time (s) in (**a**) the tail suspension test and (**b**) forced swimming test using *Aloysia gratissima* var. *gratissima* (Agg) fractions on mice (*n* = 6) after acute treatment. Im: imipramine, Hex: hexane, AcE: ethyl acetate, DCM: dichloromethane, Bu: butanol, Veh: water. One-way ANOVA and Dunnett’s post hoc test (mean ± SD); F = 44. ** *p* < 0.01; *** *p* < 0.001; **** *p* < 0.0001, as compared with control (Veh).

Following the three-treatment schedule of Agg fractions over 24 h, the ethyl acetate fraction (AggAcE) reduced immobility duration when administered at a dose of 1 mg/kg (*p* < 0.01, Figure 1a) compared with the control group treated with the vehicle (Veh = 125.2 ± 21.03) in the TST. No statistically significant differences were observed in immobility time with the hexane, dichloromethane, or butanol fractions. Imipramine, the reference antidepressant drug, also decreased immobility time (*p* < 0.0001) in the TST (Figure 1a), compared with the Veh (control) group.

The FST results also showed that the AggAcE fraction exhibited an antidepressant-like effect, which was evident when the immobility of mice was reduced with both doses (0.1 and 1 mg/kg; *p* < 0.01 and *p* < 0.001, respectively; Figure 1b) and imipramine (*p* < 0.01) compared with the control group. As expected, the animals treated with the solvent, in which each of the fractions was previously dissolved before being administered (water), showed long-duration immobility (159.1 ± 15.35).

**Figure 2:**
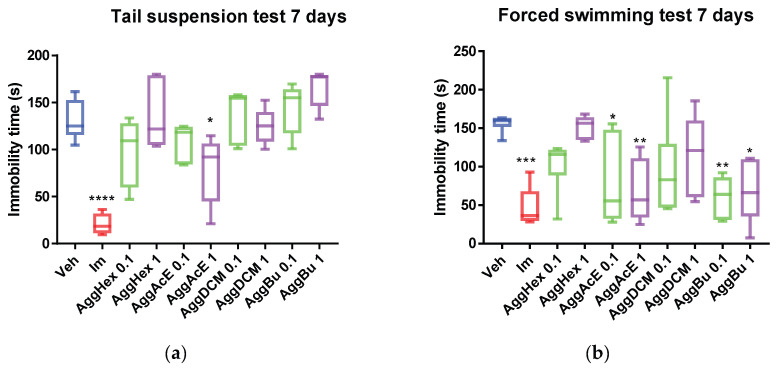
Immobility time (s) in (**a**) the tail suspension test and (**b**) forced swimming test using *Aloysia gratissima* var. *gratissima* (Agg) fractions on mice (*n* = 6) after seven days of treatment. Im: imipramine, Hex: hexane, AcE: ethyl acetate, DCM: dichloromethane, Bu: butanol, Veh: water. One-way ANOVA and Dunnett’s post hoc test (mean ± SD); F = 44. * *p* < 0.05; ** *p* < 0.01; *** *p* < 0.001, **** *p* < 0.0001, as compared with control (Veh).

We also examined the effect after 7 days of treatment. Again, the antidepressant-like effect was evident during the TST, given the decrease in immobility time with the administration of the ethyl acetate fraction (AggAcE, 1 mg/kg, *p* < 0.05, Figure 2a), as well as imipramine, when these groups were compared with the control group.

During the FST, the immobility duration was reduced by the ethyl acetate fraction (AggAcE 0.1 and 1 mg/kg; *p* < 0.05 and *p* < 0.01, respectively) and the butanol fraction (AggBu 0.1 and 1 mg/kg; *p* < 0.001 and *p* < 0.05, respectively). Imipramine was used as the positive control and markedly decreased immobility time during both the TST and FST (*p* < 0.0001 and *p* < 0.001, respectively; Figure 2b).

**Figure 3:**
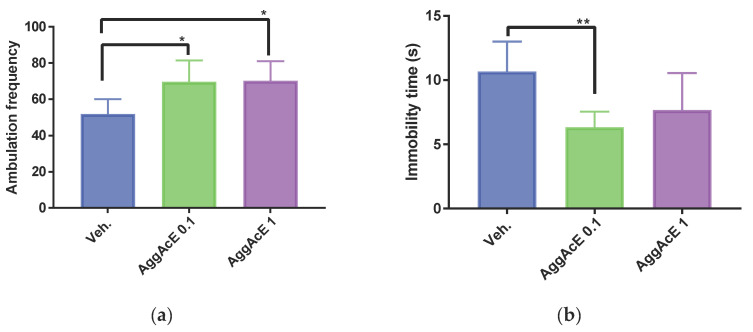

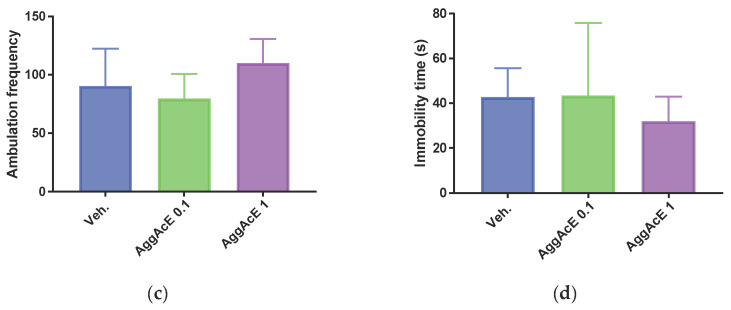
Ambulation frequency and immobility time after 24 h (**a**,**b**), and after 7 days (**c**,**d**) in open field test using the ethyl acetate fraction *Aloysia gratissima* var. *gratissima* (AggAcE) on mice (*n* = 6). Veh: water. One-way ANOVA and Dunnett’s post hoc test (mean ± SD). * *p* < 0.05; ** *p* < 0.01; as compared with control (Veh).

The effect of the AggAcE on locomotor activity in the open field test showed that after treatment for 24 h and for 7 days, neither 0.1 nor 1 mg/kg induced motor impairment (Figure 3). Compared to the vehicle, ambulation was slightly increased after 24 h (Veh: 51.83 ± 8.193; AggAcE 0.1: 69.67 ± 11.83, *p* < 0.05; AggAcE 1: 70.17 ± 10.87, *p* < 0.05). The time spent immobile with 1 mg/kg was no different from Veh (Veh: 10.67 ± 2.338; AggAcE 0.1: 6.333 ± 1.211, *p* < 0.01; AggAcE 1: 7.667 ± 2.875, *p* > 0.05). After seven days of treatment, no difference was observed between the experimental groups, either in ambulation or in the time spent immobile. After both treatment regimens, no difference in emotional behavior (rearing, grooming and defecation) of mice was observed.

### 2.2. Mechanism of Antidepressant-like Effects

Considering the results for both the acute and seven-day treatment protocols, the ethyl acetate fraction (AggAcE, 1 mg/kg) was selected to continue the analysis of the antidepressant mechanism. This fraction showed results in both tests and administration regimens.

**Figure 4:**
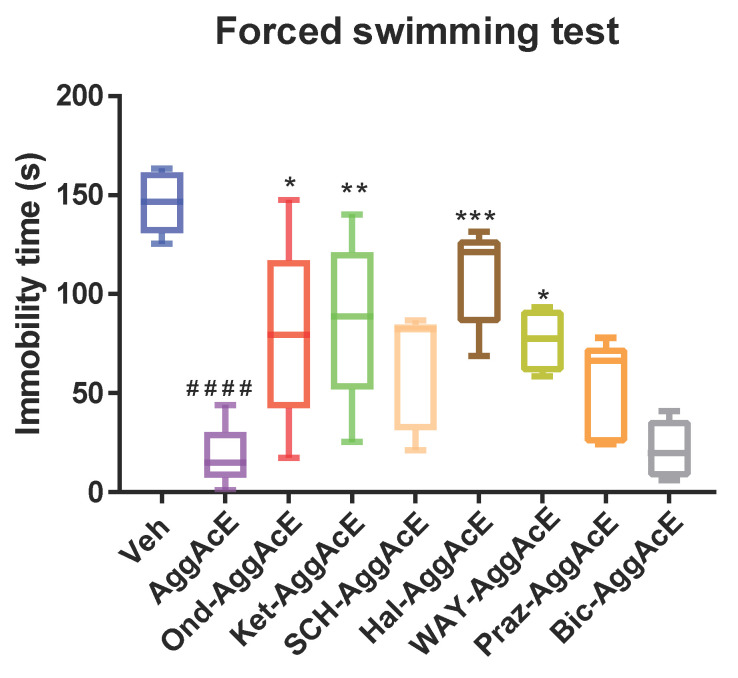
Effect of pretreating mice with ondansetron (Ond-AggAcE, 5-HT_3_ antagonist), ketanserin (Ket-AggAcE, 5-HT_2A_ antagonist), SCH23390 (SCH-AggAcE, D_1_ receptor antagonist), haloperidol (Hal-AggAcE, D_2_ receptor antagonist), WAY100635 (WAY-AggAcE, 5-HT_1A_ antagonist), prazosin (Praz-AggAcE, α_1_-adrenergic receptor antagonist), and bicuculline (Bic-AggAcE, GABA_A_ receptor antagonist) on reduced immobility time during the forced swimming test induced by the ethyl acetate fraction of *Aloysia gratissima* var. *gratissima*. The values represent the mean ± SD (*n* = 6), F = 32. Data were analyzed by one-way ANOVA, followed by Dunnett’s post hoc test. #### *p* < 0.0001, compared with the vehicle control group (Veh). * *p* < 0.05, ** *p* < 0.01, *** *p* < 0.001, compared with the AggAcE-treated group.

The effect of the serotonergic system on the antidepressant-like effects of the active fraction was tested with a 5-HT_1A_ antagonist (WAY100635), a 5-HT_2A_ antagonist (ketanserin), and a 5-HT_3_ antagonist (ondansetron). The results (Figure 4) demonstrated that pretreatment with WAY100635 prevented the reduced immobility time caused by the ethyl acetate fraction of *A. gratissima* var. *gratissima* in FST compared with the effects of AggAcE administered alone. Similarly, pretreating animals with ketanserin (5HT_2A_ antagonist) or ondansetron (5-HT_3_ antagonist) also inhibited the reduced immobility caused by AggAcE during the FST. Cotreatment using WAY100635, ketanserin, or ondansetron with AggAcE (WAY-AggAcE, *p* < 0.05; Ket-AggAcE, *p* < 0.01; Ond-AggAcE, *p* < 0.05) significantly increased the immobility time compared with the effect on the AggAcE-treated groups. The active fraction’s ability to reduce the duration of immobility was assessed, and the difference was highly significant compared with that of the control (*p* < 0.0001; *n* = 6).

To clarify the involvement of dopamine in the antidepressant-like effects, the mice were pretreated with the D_1_ receptor antagonist SCH23390 or the D_2_ receptor antagonist haloperidol. The results showed that haloperidol reversed the reduced immobility time caused by the active fraction (Hal-AggAcE, *p* < 0.001), indicating that dopaminergic mechanisms are involved in antidepressant-like effects. The SCH23390 antagonist did not affect the duration of immobility compared with AggAcE (Figure 4).

Pretreating the mice with prazosin (α_1_-adrenergic receptor antagonist) did not inhibit the reduced immobility time caused by AggAcE, ruling out the involvement of adrenergic receptor mechanisms in the antidepressant-like effects. Similarly, we found no evidence of GABAergic system involvement in the antidepressant action of Agg, considering that bicuculline was unable to reverse the reduced immobility time produced by AggAcE (Figure 4).

### 2.3. UPLC-ESI-MS Profiling of the Active Fraction of A. gratissima var. gratissima Extracts

The phytochemical profile of *Aloysia gratissima* var. *gratissima* extracts revealed several groups of metabolites, tentatively identified through their mass spectrometric and UV spectroscopic data (Table 1). The Extracted Ion Chromatograms (EICs) and the UV–vis spectra of the detected compounds from these active fractions are summarized in Figures S1 and S2, respectively.

### 2.4. ADME and Oral Bioavailability Profiles

The results obtained for the ADME property predictions of the selected compounds showed that most of them have high gastrointestinal absorption, except for 2-O-caffeoyl-hydroxymalonic acid (caffeoyl tartronic acid) and verbascoside (Table 2).

The compounds that showed a potential to permeate the blood–brain barrier were 4-hydroxybenzoic acid, 3-hydroxybenzoic acid, coumaric acid, ferulic acid, oxooctadecadienoic acid, 13-oxooctadecadienoic acid, and (6E,8Z)-5-oxooctadecadienoic acid; none of these compounds can serve as a substrate for P-glycoprotein (Table 2).

None of these compounds are inhibitors of the cytochrome P450 (CYP450) isoforms, except oxooctadecadienoic acid, 13-oxooctadecadienoic acid, and (6E,8Z)-5-oxooctadecadienoic acid, which were predicted to have inhibitory activity against the CYP1A2 and CYP2C9 isoforms. Similarly, 13-oxooctadecadienoic acid and (6E,8Z)-5-oxooctadecadienoic acid were predicted to have inhibitory activities against the CYP2D6 isoform.

Table 3 shows oral bioavailability predictions of compounds with the potential to permeate the blood–brain barrier. These compounds showed no violations of Lipinski’s rule of five and had a high bioavailability score of 0.85, which is associated with gastrointestinal properties.

### 2.5. Computational Simulations

Redocking analysis performed with ligands co-crystallized with receptors showed RMSD (*Root Mean Square Deviation*) values lower than 2 Å, indicating the validity of the scoring function and the accuracy of the simulation protocol used (Figure S3; Table 4).

**Figure 5:**
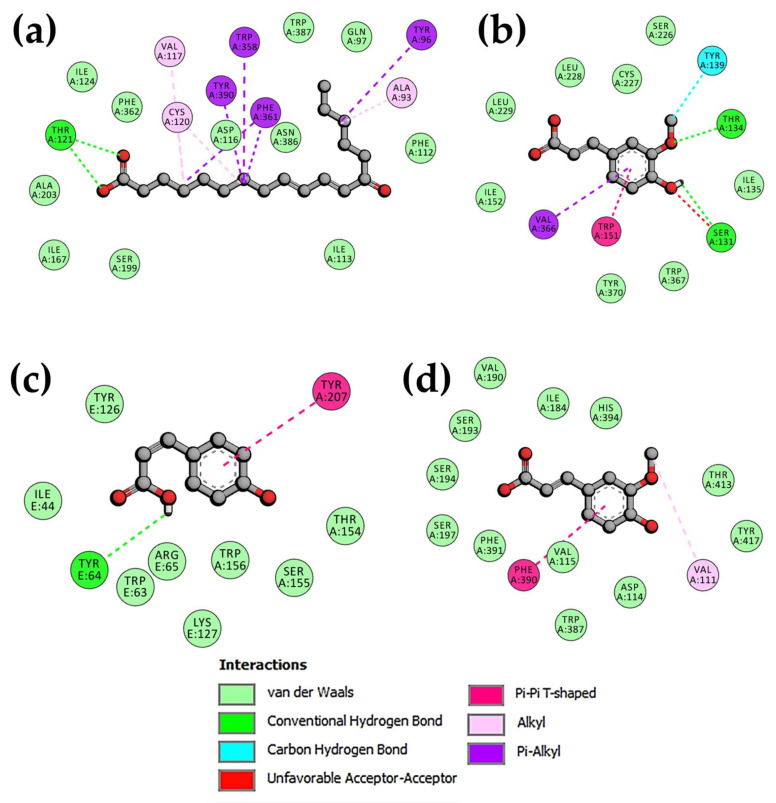
Bidimensional representation of complexes with the highest binding affinity. (**a**) 13-Oxooctadecadienoic acid:5-HT_1A_. (**b**) Ferulic acid:5-HT_2A_. (**c**) Coumaric acid:5-HT_3_. (**d**) Ferulic acid:D_2_R.

Molecular docking approximations and druggability analysis performed with receptor structures (5-HT_1A_, 5-HT_2A_, 5-HT_3_, and D_2_R) and selected compounds are presented in Table 4, Tables S2 and S3, Figure 5, Figures S4 and S5.

Among all compounds evaluated during interactions with the 5-HT_1A_ receptor, the 13-oxooctadecadienoic acid was the molecule with the highest binding affinity and the lowest ΔG and Kd_calc_ values (Table 4 and Table S2). The complex showed hydrogen bonds with Thr121; interactions between the π-orbitals of Trp358, Tyr390, Phe361, Tyr96, and the hydrocarbon region of the compound and alkyl–alkyl hydrophobic interactions with residues Val117, Cys120, and Ala93 were recorded. van der Waals interactions were also identified with residues Ile124, Phe362, Ala203, Ile167, Ser199, Ile113, Phe112, Asn386, Asp116, Trp387, and Gln97, located in the pocket binding site (Figure 5).

Ferulic acid had the lowest ΔG and Kd_calc_ values in comparison with the other phytoconstituents in interactions with 5-HT_2A_ (Table 4 and  Table S2).

The ferulic acid:5-HT_2A_ complex showed hydrogen bonds with Thr134 and Ser131 residues, and unconventional hydrogen bonds mediated by partial polarizations of carbon atoms from Tyr139 (carbon–hydrogen bond) were also detected. Additionally, π-alkyl hydrophobic interactions were recorded between π-orbitals of ferulic acid and Val366 residue, as were π-π T-shaped and π-σ hydrophobic interactions with Trp151 and the compound. van der Waals interactions were also registered with Ser226, Cys227, Leu228, Leu229, Ile152, Ile135, Trp367, and Tyr170 (Figure 5).

For the 5-HT_3_ receptor, coumaric acid was the phytoconstituent with the lowest ΔG and Kd_calc_ values (Table 4 and Table S2).

The coumaric acid:5-HT_3_ complex showed hydrogen bonds with the Tyr64 residue of chain E and π-π T-shaped hydrophobic interactions with the Tyr207 residue of chain A. van der Waals interactions were also detected with residues Tyr126, Ile44, Arg65, Trp63, and Lys127 of chain E and residues Trp156, Ser155, and Thr154 of chain A, also located in the pocket binding site (Figure 5).

For the D_2_R receptor, ferulic acid showed the lowest ΔG and Kd_calc_ values (Table 4 and Table S2).

The ferulic acid:D_2_R complex showed only hydrophobic interactions, including alkyl–alkyl interactions with Val111, as well as interactions between the π-orbitals (π-π T-shaped) of Phe390 and the aromatic ring of ferulic acid. In addition, van der Waals interactions were recorded with Val190, Ile184, His394, Ser193, Ser194, Ser197, Phe391, Val115, Asp114, Trp387, Tyr417, and Thr413 (Figure 5).

## 3. Discussion

Rural communities in developing countries rely on traditional herbal medicines to treat their illnesses [8]. Several have been shown to play a therapeutic role in treating depression at a lower cost, and, in general, the adverse effects of using medicinal plants are lower [14]. Conventional antidepressants used as first-line therapy for depression include serotonin–norepinephrine reuptake inhibitors, selective serotonin reuptake inhibitors, serotonin modulators, and atypical antidepressants [5]. Among the disadvantages of these first-line drugs are cholinergic effects and weight gain. Therefore, research into new ways to treat depression is needed [15,16,17].

The two most common animal models for evaluating the action of samples with a potentially antidepressant effect are the tail suspension test (TST) and the forced swimming test (FST). The TST involves suspending mice by their tails with adhesive tape; in the FST, mice are placed in a cylinder in an inescapable situation. In both tests, the animals are observed for six minutes. Their escape-related mobility behavior is measured during the last four minutes. In both tests, an antidepressant-like effect induced a decrease in immobility time compared with a control group [18,19].

In line with our previous research on aerial parts [13], the present findings support the antidepressant-like potential of the ethyl acetate fraction of *Aloysia gratissima* var. *gratissima* (AggAcE), as evidenced by reduced immobility time after oral treatment in both the TST and the FST. This effect was comparable to that of the standard antidepressant imipramine. When mice are subjected to stressful situations, they become immobile after a brief period of struggling, as was the case in the experiments performed here; this can be interpreted as a reflection of behavioral despair. Therefore, the TST and FST are valuable tools in the preclinical study of potential antidepressants [18,19]. Our results demonstrate the ability of AggAcE in the reversion of behavioral despair in mice orally pretreated with both 0.1 and 1 mg/kg (Figure 1 and Figure 2). Furthermore, 1 mg/kg of AggAcE (*p* < 0.001 compared with the control group) exhibited a stronger effect than imipramine (32 mg/Kg, i.p.; *p* < 0.01 compared with the control) during the forced swimming test after treating mice three times over 24 h. This is interesting given that plant extracts (or fractions) contain complex mixtures of phytochemicals that vary in their abundance, often working synergistically [20]. Furthermore, in the open field trial (on day 1, and on day 7) with the ethyl acetate fraction, it has been shown that it does not induce sedation or motor impairment in mice treated with orally administered doses of 0.1 and 1 mg/kg, as no difference was observed in the time spent immobile compared to the control group. Previous studies have shown that the crude extract of Agg does not induce sedation or motor impairment in mice treated with orally administered doses between 50 and 200 mg/kg [21]. Additionally, in the FST with the ethyl acetate fraction, no motor impairment was observed, as there was no reduction in active fighting (kicking or climbing) nor an early increase in immobility (floating), unlike the control group [10,18].

Butanol fraction also exhibited an antidepressant-like effect, but the reduced immobility time was evident only after seven days of treatment, and only during the FST. No effect was detected during the TST when this fraction was administered, precluding further studies with the butanol fraction. This result could be due to the amount of active ingredients present in the butanol fraction, relative to the amount present in the ethyl acetate fraction, where a slight antidepressant-like effect was observed in the caudal suspension test. Notably, the mice treated with imipramine showed an antidepressant-like effect, that is, a significant reduction in immobility time during both the TST and the FST (*p* < 0.0001 and *p* < 0.001, respectively), thus validating the method. Considering these results, to determine the mechanism of action, we tested the interaction between the antagonist and ethyl acetate fraction (AggAcE).

The monoaminergic system is one of the most important targets in the pathophysiology and treatment of depression [22]. Thus, our study explored the influence of this system on the antidepressant-like effect of AggAcE. We explored the serotonergic system’s involvement in antidepressant-like effects with the 5-HT_1A_ antagonist (WAY100635), 5-HT_2A_ antagonist (ketanserin), and 5-HT_3_ antagonist (ondansetron). The involvement of the dopamine system was tested with D_1_ receptor antagonist SCH23390 and the D_2_ receptor antagonist haloperidol. In addition, the involvement of the adrenergic system was assayed with prazosin (α_1_-adrenergic receptor antagonist). Furthermore, we tested the GABAergic system’s involvement in the antidepressant action of Agg using bicuculline, considering the growing body of evidence pointing to an association between major depressive disorders and diverse types of GABAergic deficits [23,24].

The pathophysiological basis of depression is due, at least in part, to a decrease in the levels of serotonin, norepinephrine, and/or dopamine in the central nervous system. The mechanism of action of antidepressants currently used in the treatment increases the levels of these neurotransmitters in the brain [5]. Most antidepressant drugs in clinical use promote an increase in 5-HT availability, directly affecting serotonin turnover and reuptake and interacting with the 5-HT_1A_ and 5-HT_2_ receptors [25]. Dysregulation of brain dopamine (DA), primarily in mesolimbic and mesocortical neuronal systems, influences the pathophysiology of depression and offers new potential targets for drug development focused on DA-modulated micro-circuits. Pharmacological interventions that block or reduce DA worsen depression in humans. Furthermore, antidepressant drugs such as imipramine or amitriptyline have been found to enhance DA mechanisms in rats [8,26]. One hour after oral AggAcE administration, reduced immobility time was evident. However, with ondansetron, ketanserin, WAY100635, and haloperidol, this reduction was reversed. Animals pretreated with these antagonists exhibited significant differences compared with the AggAcE-treated group, providing evidence that the effects of AggAcE are probably mediated by an interaction with the serotonergic and dopaminergic systems.

Nevertheless, to firmly establish the mechanisms involved, more studies are needed. Evidence of the mechanisms involved in depression includes hypothalamus–pituitary axis dysregulation, a rise in oxidative stress and inflammatory mediators, reduced neurotrophic factors, and hypofunction of N-methyl-D-aspartate receptors [8]. For example, Zeni et al. studied the oil of *A. gratissima*, a variety of Agg, and demonstrated that antidepressant-like activity occurs through the inhibition of N-methyl-D-aspartate (NMDA) receptors [27]. Another difference is that these authors tested the activity in an aqueous extract, a more complex matrix of metabolites; they associated the antidepressant effect with interactions with the serotonergic, noradrenergic, and dopaminergic systems [28]. In our research, the active fraction did have an effect on the adrenergic system. Moroni et al. described a complex of four *A. gratissima* varieties, contributing to variation in the composition of secondary metabolites and, therefore, mechanisms [29].

In our study, phenolic acids and related derivatives were among the most abundant metabolites. Compounds **1** and **2** were consistent with hydroxybenzoic acid, showing [M−H]^−^ ions at *m*/*z* 137 and UV absorption maxima near 255 nm [30]. Compound **3** was identified as coumaric acid, supported by its [M−H]^−^ ion at *m*/*z* 163 and absorption at 307 nm [30,31]. Ferulic acid (compound **6**) was identified by its [M−H]^−^ ion at *m*/*z* 193 and a diagnostic band at 325 nm [31], while methylferulic acid (compound **10**) showed a pseudomolecular ion at *m*/*z* 207 and a UV maximum at 324 nm [32]. Additionally, compound **4** was tentatively identified as 2-O-caffeoyl-hydroxymalonic acid, considering its [M−H]^−^ ion at *m*/*z* 281 and caffeate-type absorption at 329 nm [33].

Within the phenylethanoid glycosides, verbascoside (compound **5**) was identified by its pseudomolecular ion at *m*/*z* 623 and a characteristic UV absorption maximum at 329 nm [31].

Flavonoids and methylated flavones were also abundant. Peak 8 ([M−H]^−^ at *m*/*z* 373) was consistent with methylsudachitin [34]. Methoxyapigenin (compound **13**) was supported by its pseudomolecular ion at *m*/*z* 299 and UV maxima at 331 and 271 nm [31]. Hydroxy-di-O-methylluteolin (compound **14**) shows a [M−H]^−^ ion at *m*/*z* 329 [34], while compound **15**, with [M−H]^−^ at *m*/*z* 313 and maxima at 343 and 271 nm, was consistent with dihydroxy-dimethoxyflavone [31]. Compound **16** ([M−H]^−^ at *m*/*z* 343) was tentatively identified as dihydroxy-trimethoxyflavone [35]. Methyl apigenin (compound **17**) was supported by its [M−H]^−^ ion at *m*/*z* 283 and UV maxima at 342 and 270 nm. Peak 12, with [M−H]^−^ at *m*/*z* 327 and absorption at 309 nm, was consistent with a coumaroyl derivative.

Some terpenoid derivatives were detected, including compounds **7** and **19**, both of which exhibited [M+H]^+^ ions at *m*/*z* 337, in agreement with hydroxylated methylhoffmanniaketone isomers [36]. The only fatty acid derivative was compound **20**, with [M−H]^−^ at *m*/*z* 293, tentatively identified as oxooctadecadienoic acid [34].

Compounds **9** and **11**, despite weak UV absorption, were observed with significant intensity. Compound **9** ([M+H]^+^ at *m*/*z* 341) was compatible with trijuganone C [37], while the 114 Da difference with compound **11** ([M+H]^+^ at *m*/*z* 454) suggested a glutaryl derivative, pending further confirmation. Similarly, compounds **18** and **21** ([M−H]^−^ at *m*/*z* 267 and 284; weak UV absorption near 300 nm) were consistent with ketonic structures but remain uncharacterized.

Several of these metabolites—including diterpenes, coumaric acid, verbascoside, ferulic acid, and methylated flavonoids—have been previously reported in *A. gratissima* [31] and *A. gratissima* var. *gratissima* leaves [13], supporting the current findings. Moreover, caffeoyl tartronic acid has been described in diverse plant species such as *Nepeta cataria*, *Glechoma hederacea*, *Vigna radiata*, and *Chondrilla juncea* [33,38,39,40]; to the best of our knowledge, this is the first report of its occurrence in *A. gratissima* var. *gratissima*. Overall, the detection of phenylethanoids, diterpenes, monoterpenes, hydroxycinnamic acids, and methylated flavonoids reflects the chemical diversity previously described in the Verbenaceae family, particularly within *Aloysia* and *Lippia* [13,31,41,42]. Importantly, this study provides the first chemical characterization of active fractions of *A. gratissima* var. *gratissima* associated with antidepressant-like effects. Nevertheless, further work is still needed, including comprehensive spectroscopic and spectrometric analyses to confirm compound identities and strengthen mechanistic interpretations.

Predictions regarding the blood–brain barrier permeant activity of compounds are consistent with previous reports that highlight the presence of 4-hydroxybenzoic acid in the mammalian brain as a rapid product of the oxidative metabolism of the 4-hydroxybenzaldehyde compound [43,44]. The presence of 3-hydroxybenzoic acid has also been reported in human cerebrospinal fluid [45].

In the same way, coumaric acid, ferulic acid, and an isomer of oxooctadecadienoic acid (9-oxo-octadecadienoic acid) have also been described in previous studies as molecules with blood–brain barrier permeant activities [46,47]. However, methylferulic acid has been reported to have very low blood–brain barrier permeant potential [48].

In silico predictions also revealed some compounds (oxooctadecadienoic acid, 13-oxooctadecadienoic acid, and (6E,8Z)-5-oxooctadecadienoic acid) with potential inhibitory activities in cytochrome P450 isoforms (CYP1A2, CYP2D6, and CYP2C9). However, no studies have shown the inhibitory activities of these compounds on any of the CYP450 isoforms. These compounds, considered oxylipins, have been reported as products of linoleic acid metabolism mediated by cytochrome P450 isoforms, and they are thus considered potential substrates of these enzymes [49].

Molecular docking simulations with selected phytoconstituents showed similar binding affinities to the molecular structures of all receptors evaluated in this study (5-HT_1A_, 5-HT_2A_, 5-HT_3_, and D_2_R), with approximations agreeing with the ΔG values (−7.1 to −4.6 kcal/mol) and calculated Kd_calc_ values (Table S2). However, these binding affinities were lower than those registered with standard inhibitors (Table 4 and Table S2).

Notably, the binding site of 13-oxooctadecadienoic acid in the 5-HT_1A_ receptor topologically corresponds to the interaction region of the standard inhibitor Way100635 used in this study, which, in turn, is located within an identified druggable site (Figure S4). This site showed a druggability score of 0.82, indicating a high pharmacological potential for drug design studies [50] (Figure S5; Table S3).

Active residues identified in the 5-HT_1A_:13-oxooctadecadienoic acid complex were also found at the identified druggable site. Furthermore, residues such as Cys120, Asp116, Thr121, Phe361, and Tyr390 play fundamental roles in ligand recognition, and residues Asp116 and Phe361 are essential for the stability of the protein [51].

The ferulic acid binding site topologically colocalizes with the inhibitor ketanserin. Likewise, this region is located in an identified druggable site (Figure S4), which showed a high score (0.99), indicating a high pharmacophore capacity for drug design [52] (Figure S5; Table S3).

Previous studies have shown that Trp151 and Cys227 residues play a very important role in stabilizing ligands in the receptor (5-HT_2A_) by establishing interactions mediated by π-orbitals present in aromatic rings of the residue side chain in the case of Trp151, and hydrogen bonds and hydrophobic interactions in the case of Cys227 [52]. Ser131 and Thr134 residues actively participate in the interactions with ligands and receptor inhibitors due to their ability to maintain hydrogen bond interactions because of low hydrophobicity, the polarity of the side chain, and the presence of hydroxyl functional groups [53].

Coumaric acid has interaction affinity with the same binding site as ondansetron (standard inhibitor) and is a neuroprotector compound [46]. This region was also identified as a druggable site with a score of 0.82, indicative of pharmacophoric suitability [50] (Figure S5; Table S3).

In this study, the active residues that interacted with coumaric acid were also located at the predicted druggable site. Earlier investigations emphasized the functional relevance of Trp63, Trp156, Tyr126, Tyr207, and Tyr64 in ligand/neurotransmitter recognition due to the presence of an aromatic ring in the side chain of these residues; this directly contributes to the stability of ligand/neurotransmitter interactions [54].

Similarly, ferulic acid interacts with the D_2_R receptor in the same region as haloperidol (standard inhibitor), which corresponds to a druggable site identified in the receptor (Figures S4 and S5). The registered druggable site showed a score of 0.81 and a hydrophobicity ratio of 43%, supporting its relevance for structure-based drug development strategies [52] (Figure S5; Table S3).

Previous research has reported that Val11 and Phe390 are essential for ligand interactions, as well as for complex stabilization and D_2_R receptor activation. The interactions of ligand molecules with Ile184 and Asp114 are also crucial for antagonist/agonist activities [55].

The binding affinity of compounds for the same interaction sites of the standard inhibitors in receptors (Way100635, ketanserin, ondansetron, and haloperidol), based on computational approximations, suggest a potential competitive activity for the receptor-predicted druggable site; however, experimental validations using techniques such as radioligand binding assays or CETSAs (Cellular Thermal Shift Assays) with isolated compounds are necessary [56].

Despite AlphaFold’s predictive accuracy in protein structural modeling, challenges persist in accurately modeling G-Protein-Coupled Receptors (GPCRs) due to their intrinsic conformational flexibility. Despite these limitations, this research opted to use structures modeled by AlphaFold (5-HT_2A_ and D_2_R receptors) due to the absence of crystallized structures and their prior application in related computational investigations [57,58].

## 4. Materials and Methods

### 4.1. Reagents and Chemicals

All chemicals used in this study were analytical grade. Imipramine hydrochloride and sodium chloride were obtained from Wako Pure Chemical Industries Ltd. (Osaka, Japan); ketanserin, WAY100635, ondansetron, SCH233390, haloperidol, prazosin, bicuculline, and DMSO were purchased from Sigma-Aldrich, St. Louis, Missouri, USA. Ethanol for pharmaceutical use was locally purchased. Hexane, dichloromethane, ethyl acetate, and butanol came from Scharlau, Barcelona, Spain. All reagents used for the UPLC analysis were LC grade.

### 4.2. Animals

Male Swiss albino mice in a weight range of 25 to 35 g were used. The experimental animals came from the bioterium of the Faculty of Chemical Sciences, UNA. Until use, they were kept in cages, in standard light/dark cycle conditions, at a room temperature of 20–25 °C, and at relative room humidity (70–80%). Mice were provided with fresh water and food daily (standard pellets). On the day of the experiment, the mice were weighed and properly identified. They were then taken to the testing laboratory at least one hour before the experiments began. All experiments were carried out between 8:00 and 11:00 A.M.

### 4.3. Plant Material, Extraction and Fractionation

*Aloysia gratissima* var. *gratissima* (Gillies & Hook. ex-Hook.) Tronc. aerial parts were collected from the Garden of Acclimatization of Native and Medicinal Plants, Facultad de Ciencias Químicas (FCQ), Universidad Nacional de Asunción (San Lorenzo, Paraguay), and identified (Y. González 197/2024) by researchers from the Department of Botany—FCQ. Ethanol extract was prepared with freshly collected, dried (oven, 40 °C), and powdered material by sonication. The solvent was removed, and the residue was partitioned into successive fractions with hexane, dichloromethane, ethyl acetate, and butanol.

### 4.4. Tail Suspension Test of Mice

The mice were tested to evaluate the possible antidepressant-like effect of fractions using the tail suspension test (TST) [13,19]. For administration to the animals, each fraction was pre-dissolved in water on the day of administration. The effects of the hexane, dichloromethane, ethyl acetate, and butanol fractions of *A. gratissima* var. *gratissima* (Agg) were analyzed. For this purpose, the mice were treated three times over a 24 h period with 0.1 or 1 mg/kg of Agg fractions. In the other experimental groups, the treatment was administered daily for seven days. The selected doses were based on our previous results with Agg ethanol extract, where an antidepressant-like effect was observed at all doses analyzed, the lowest being 50 mg/kg (*p* < 0.0001). Therefore, we used at least one-tenth of the effective dose (50 mg/kg) [13]. The administration schedule was decided considering our previous research with the same and other plants [13] and considering that antidepressant treatment in clinical practice usually has a delayed effect. Thus, the animals (*n* = 6) in the first administration regimen were treated with the vehicle in which the fractions were dissolved (water; 0.1 mL/10 g of weight, orally), as well as doses of the hexane fraction of *A. gratissima* var. *gratissima* (AggHex, 0.1 and 1 mg/kg, orally), the dichloromethane fraction (AggDCM, 0.1 and 1 mg/kg, orally), the ethyl acetate fraction (AggAcE, 0.1 and 1 mg/kg, orally), the butanol fraction (AggBu, 0.1 and 1 mg/kg, orally), and imipramine (Im 32 mg/kg, intraperitoneal route). In the first set of experiments, this treatment was administered 24, 18, and 1 h before the mice were individually subjected to the tail suspension test, except for the imipramine group, where the last dose was 20 min before the TST assay. In the second case (7 days), the animals were treated for 7 consecutive days and, one hour after the last administration, they were individually subjected to the same tail suspension procedure. The imipramine-treated group was subjected to the FST 20 min after the last treatment [19]. Each 6 min session was videotaped, and subsequent analysis was performed with the EthoVision software; the last 4 min were counted for analysis. Animal movement was detected with automated 3-point software (EthoVision XT17, Version essential, multiple arena module, Noldus, Wageningen, The Netherlands). For analysis, each of the six arenas was divided equally. The EthoVision XT17 software calculated the time spent immobile.

### 4.5. Forced Swimming Test in Mice

Immediately after the mice passed the TST, they were subjected to the forced swimming test (FST). This procedure has been used previously in our research group, thus requiring fewer animals [13]. Although the previous test induces a stressful situation, which could affect the interpretation of the results in the forced swim test and constitute a limitation of this study, we have followed the same procedure in previous studies to reduce the number of experimental animals used [13]. To minimize interpretation errors and verify the appearance of other effects that might confound the behavioral assessment, control groups (treated with water, the vehicle in which the samples were dissolved for administration) and a group treated with imipramine—the reference antidepressant—were also included. Both groups yielded expected results during the forced swim test; that is, the control group showed typical behavioral despair, resulting in a long period of immobility (depressive-like state), while the group treated with imipramine experienced a reduced immobility time. The mice were individually introduced into a transparent cylinder (25 cm high and 15 cm in diameter) containing 12.5 cm of water maintained between 23 and 24 °C. Each 6 min session was videotaped; subsequent analysis was performed with the EthoVision software, and data were tabulated. The immobility time in the last four minutes was measured [18,19]. In all cases, the cylinder was cleaned with 10% ethanol solution before being refilled with clean water.

### 4.6. Open Field Test

The open field test was performed one hour after oral treatment with vehicle (Veh, 0.1 mL/10 g body weight) or AggAcE (0.1 and 1 mg/kg), after 24 h of treatment and after 7 days of treatment. Mice were placed individually in the central part of the box arena and allowed to freely explore the box for five minutes. The parameters recorded were the number of rearing, number of defecations, number of crossings (spontaneous locomotor activity), and the time spent immobile. Motor impairment is associated with decreased exploration activity and significant reductions in movement. Emotional involvement is evidenced by increased rearing and defecation. The apparatus was cleaned with ethanol solution (10%) before placing the next mouse, and the test was carried out in a light-controlled (red light, 15 W) room [21,59].

### 4.7. Mechanism Involved in the Antidepressant-like Effects of Active Fraction of Aloysia gratissima var. gratissima

The mechanism of action involved in the antidepressant activity of the active fraction was determined using several antagonists. Mice were divided into 9 groups, with 6 animals in each group [59,60]. To assess the involvement of the serotonergic system in the antidepressant-like effects of the active fraction, mice were pretreated with WAY100635 (a 5HT_1A_ antagonist; 0.1 mg/kg i.p.), ketanserin (a 5HT_2A_ antagonist; 1 mg/kg i.p.), or ondansetron (a 5-HT_3_ antagonist; 1 mg/kg i.p.). The dopaminergic system’s involvement was also assessed by pretreating mice with either SCH23390 (a D_1_ antagonist; 0.05 mg/kg, i.p.) or haloperidol (a D_2_ antagonist; 0.2 mg/kg, i.p.). Similarly, to assess the adrenergic system, mice were treated with prazosin (an α1-adrenergic antagonist, 1 mg/kg, i.p.). Finally, the GABAergic system’s involvement was investigated by treating mice with bicuculline (a GABAergic antagonist; 2 mg/kg, i.p.).

Mice were pretreated with the antagonist (WAY100635, ketanserin, ondansetron, SCH23390, haloperidol, prazosin, or bicuculline). All agonists were dissolved in saline solution containing 1% DMSO [59,60]. After 25 min, the animals in each subgroup received the ethyl acetate fraction orally (AggAcE, 1 mg/kg). After 35 min of treatment with the active fraction, the animals underwent the FST, as previously described. Additionally, the control group received water, and another group was treated with the active fraction (ethyl acetate, AggAcE; 1 mg/kg, p.o.).

### 4.8. UPLC-ESI-MS Profiling of the Active Fractions of A. gratissima Extract

Analyses were performed using a Waters Acquity UPLC system (Milford, CT, USA) equipped with an Acquity PDA eλ detector and coupled with a Xevo TQD triple quadrupole mass spectrometer (QqQ-MS) with an electrospray ionization (ESI) source. Chromatographic separations were achieved on a Phenomenex KINETEX core–shell EVO-C18 column (2.1 × 100 mm^2^, 1.7 µm) operated at a flow rate of 0.3 mL/min. The column temperature was maintained at 40 °C, and the injection volume was 10 µL. The mobile phase consisted of water (phase A) and acetonitrile (phase B), both containing 0.1% formic acid and 10 mM ammonium formate. Gradient elution was applied as follows: 0–0.7 min, 90% A; 0.7–2.0 min, 90–80% A; 2.0–6.0 min, 80–45% A; 6.0–9.0 min, 45–0% A; 9.0–11.0 min, 0–60% A; 11.0–14.0 min, 60–90% A. This was followed by re-equilibration at 90% A for 15.0 min. Extracts (5 mg/mL) were dissolved in a 1:1 mixture of water and acetonitrile and filtered through 0.22 µm nylon syringe filters prior to injection. UV monitoring was conducted at 280, 320, and 500 nm, and PDA spectra were recorded from 200 to 500 nm for peak characterization. Mass spectrometric data were acquired in full-scan mode (*m*/*z* 100–1000) under both positive and negative ionization. Nitrogen was used as a nebulizing and drying gas, while argon served as the collision gas. The MS parameters were as follows: capillary voltage, 3.5 kV; source temperature, 150 °C; desolvation temperature, 350 °C; cone voltage, 30 V; cone gas flow, 80 L/h; and desolvation gas flow, 900 L/h. Instrument control and data acquisition were managed using Waters MassLynx v4.1.

Compounds were tentatively identified based on their pseudomolecular ions, UV–vis absorption maxima, and comparison with previously published data for *Aloysia* or other species when available.

### 4.9. Pharmacokinetic and Oral Bioavailability Predictions

Pharmacokinetic predictions (ADME: Absorption, Distribution, Metabolism, and Excretion) were performed with compounds identified in this study and that are available in the PubChem database. Some compounds have more than one isomer, so all were considered equally for in silico study (Table S1) [61]. Lipinski’s rule of five was performed for oral bioavailability prediction (molecular weight less than 500 g/mol, number of H-bond donors less than 5, number of H-bond acceptors less than 10, and water–octanol partition coefficient value less than 5) only in compounds with the ability to permeate the blood–brain barrier [62]. These analyses were performed using the SwissADME tool [63].

### 4.10. Selection of Molecular Targets and Druggability Analysis

The selected molecular targets were the 5-HT_1A_ receptor (PDB: 8JT6), the 5-HT_2A_ receptor (AlphaFold Database: AF-P35363-F1), the 5-HT_3_ receptor (PDB: 4PIR), and the D_2_R receptor (AlphaFold Database: AF-P61168-F1), which are related to the serotonergic and dopaminergic pathways [64,65].

The molecular structures of these targets were obtained from the Protein Data Bank and AlphaFold Database. The structural analysis of druggable sites was carried out using Gaussian difference methods as a filter for the characterization (dimensions, composition, and druggability score) of pocket binding sites in the selected receptors, using the DoGSiteScorer tool [50,66].

### 4.11. Molecular Docking Simulations

Semiflexible docking simulations were performed using the chemical structure of compounds with the prediction potential to permeate the blood–brain barrier and the selected molecular targets. Energy minimization of the molecular structures was performed using the Merck Molecular Force Field 94 (MMFF94) algorithm, 50,000 steps of conjugate gradients, and an energy convergence of 0.01 kcal/mol.Å. Hydrogen atoms were present at a physiological pH of 7.2, and partial charges were added using Avogadro v.1 [67].

Simulations were performed using dimensions of a docking box of 53 × 60 × 80 Å^3^ for the 5-HT_1A_ receptor, 146 × 69 × 131 Å^3^ for the 5-HT_2A_ receptor, 81 × 103 × 157 Å^3^ for the 5-HT_3_ receptor, and 158 × 87 × 122 Å^3^ for the D_2_R receptor, using AutoDock Vina v.1 [68]. Binding affinity was approximated by calculating the free binding energy (ΔG) and the dissociation constant (Kd_calc_) from the ∆G values [69].

$$Kdcalc=e^{\left(\frac{\Delta G\times 1000}{RT}\right)}$$

*T* is the temperature of 310 K (37 °C); the *R* constant is 1.987207 cal/mol.K.

Analysis of the complexes was carried out using Discovery Studio Visualizer v.20 (Dassault Systèmes BIOVIA, San Diego, CA, USA). The score and the molecular docking protocol were validated using redocking ligands co-crystallized with the 5-HT_1A_ (PDB: 7E2Y), 5HT_2A_ (PDB: 8UWL), 5-HT_3_ (PDB: 6Y1Z), and D_2_R (PDB: 8IRS) receptors using the same simulation protocols.

### 4.12. Statistical Analysis

The data obtained from the study groups are expressed as means ± standard deviations. Statistical analysis was performed using GraphPad Prism 7.0 (La Jolla, CA, USA). Normality and homogeneity of variances were checked using the Shapiro–Wilk and Levene tests. One-way ANOVA followed by Dunnett’s multiple comparison test was performed. A level of *p* < 0.05 was considered statistically significant. We used GraphPad Prism version 7.0 for Windows, GraphPad Software (www.graphpad.com).

### 4.13. Ethical Issues

All experiments were carried out in accordance with the standards established by the European Community Ethics Commission. The handling of the animals was carried out with standardized procedures, and the basic rule was that “all treated animals must be euthanized.” The protocol was approved by the Research Ethics Committee of the Facultad de Ciencias Químicas, UNA (Code CEI 944/2022). The minimum number of animals and the shortest duration of observation required to obtain consistent data were used. Each animal was used once. At the end of the study, the mice were subjected to an end-of-cycle procedure following animal welfare regulations [70].

## 5. Conclusions

The results showed that *Aloysia gratissima* var. *gratissima* retains antidepressant-like activity in its ethyl acetate and butanol fractions. The chemical composition of both fractions was similar. The ethyl acetate fraction showed an antidepressant-like effect in both behavioral assays, which appears to be comparable to that of imipramine, without altering the locomotion of the treated animals. The mechanism by which the ethyl acetate fraction reduces immobility in mice appears to be mediated, at least in part, by serotoninergic and dopaminergic systems. Further studies are required to fully understand the molecular mechanism of action, as well as the interference of other factors in the interpretation of the data. Forced exposure can induce stressful situations and discomfort in animals, which could lead to errors in interpreting the results. Therefore, it is important to verify the effect of a control group and the use of a sensitive reference antidepressant in both behavioral trials. In the present study, the expected results were also obtained with the comparison groups. In silico approaches suggest that, among compounds with predictive blood barrier permeant potential, 13-oxooctadecadienoic acid, ferulic acid, and coumaric acid have binding affinities to the druggable site of the serotonergic and dopaminergic receptors; however, these suggestions require experimental confirmation. This study represents the first chemical analysis of the active fractions of *Aloysia gratissima* var. *gratissima* linked to antidepressant-like activity. These findings contribute to our understanding of the phytochemical basis of its bioactivity and will generate hypotheses for receptor-level interactions. Future work should include fully spectroscopic and spectrometric confirmation of compound identities to consolidate mechanistic interpretations.

## Figures and Tables

**Table 1:** Tentative identification of compounds detected in *A. gratissima* var. *gratissima* ethyl acetate (EA) and butanol (B) extracts through UPLC-DAD-ESI-MS.

Peak	Rt (min)	UVmax (nm)	[M−H]^−^	[M+H]^+^	Tentative Identification	EA	B
1	1.59–1.71	254	136.58		Hydroxybenzoic acid	X	X
2	2.98		136.58		Hydroxybenzoic acid 2	X	X
3	3.05–3.18	307, 295sh	162.87		Coumaric acid	X	X
4	3.46–3.51	321, 296sh	280.82		2-O-caffeoyl-hydroxymalonic acid (caffeoyl tartronic acid)	X	X
5	3.87–4.01	329, 296sh	622.68		Verbascoside	X	X
6	4.12–4.29	325, 286sh	192.82		Ferulic acid	X	X
7	4.42–4.49	245, 305		337.23	Hydroxymethylhoffmanniaketone	X	X
8	4.52		372.52		Methylsudachitin	X	X
9	4.89–4.92	275		340.37	Unknown	X	
10	4.95–5.05	324, 295sh	207.01		Methyl ferulic acid	X	X
11	5.39			453.49	Unknown	X	X
12	5.56–5.69	310, 295sh	327.21		Coumaroyl derivative	X	X
13	5.76–5.82	331, 270	298.98		Methoxyapigenin	X	X
14	5.99		329.00		Hydroxy-di-O-methylluteolin	X	X
15	6.30–6.40	343, 275	312.52		Dihydroxy-dimethoxyflavone	X	X
16	6.36–6.51	330, 281	342.61		Dihydroxy-trimethoxy flavone	X	X
17	6.84–6.91	342, 270	282.86		Methyl apigenin	X	X
18	7.74–8.03			267.11	Unknown	X	X
19	7.91–7.93	222		337.31	Hydroxymethylhoffmanniaketone isomer	X	X
20	7.89		293.47		Oxooctadeca-dienoic acid	X	X
21	8.79			284.29	Unknown	X	X

**Table 2:** ADME prediction of selected compounds for in silico analysis.

Compounds	GI-A	BBB-P	Pgp-S	Inhibition				
				CYP1A2	CYP2C19	CYP2C9	CYP2D6	CYP3A4
4-Hydroxybenzoic acid	High	Yes	No	No	No	No	No	No
3-Hydroxybenzoic acid	High	Yes	No	No	No	No	No	No
Coumaric acid	High	Yes	No	No	No	No	No	No
2-O-caffeoyl-hydroxymalonic acid (caffeoyl tartronic acid)	Low	No	No	No	No	No	No	No
Verbascoside	Low	No	Yes	No	No	No	No	No
Ferulic acid	High	Yes	No	No	No	No	No	No
Hydroxymethylhoffmanniaketone	High	No	Yes	No	No	No	No	No
Methylsudachitin	High	No	No	Yes	No	Yes	No	Yes
Methylferulic acid	High	Yes	No	No	No	No	No	No
3′-Methoxyapigenin	High	No	No	Yes	No	Yes	Yes	Yes
6-methoxyapigenin	High	No	No	Yes	No	No	Yes	Yes
3-Methoxyapigenin	High	No	No	Yes	No	No	Yes	Yes
8-Methoxyapigenin	High	No	No	Yes	No	No	Yes	Yes
Hydroxy-di-O-methylluteolin	High	No	No	Yes	No	Yes	Yes	Yes
Dihydroxy-dimethoxyflavone	High	No	No	Yes	No	Yes	Yes	Yes
Dihydroxy-trimethoxyflavone	High	No	No	Yes	No	Yes	Yes	Yes
Methylapigenin	High	No	No	Yes	No	No	Yes	Yes
Hydroxymethylhoffmanniaketone isomer	High	No	Yes	No	No	No	No	No
Oxooctadecadienoic acid	High	Yes	No	Yes	No	Yes	No	No
13-Oxooctadecadienoic acid	High	Yes	No	Yes	No	Yes	Yes	No
(6E,8Z)-5-oxooctadecadienoic acid	High	Yes	No	Yes	No	Yes	Yes	No

GI-A: gastrointestinal absorption, BBB-P: blood–brain barrier permeant, Pgp-S: P-glycoprotein substrate.

**Table 3:** Oral bioavailability of selected BBB-permeant compounds.

Compounds	Molecular Weight (g/mol)	H-Bond Acceptors	H-Bond Donors	LogP	Infractions	Bioavailability Score
4-Hydroxybenzoic acid	138.12	3	2	1.05	0	0.85
3-Hydroxybenzoic acid	138.12	3	2	1.03	0	0.85
Coumaric acid	164.16	3	2	1.26	0	0.85
Ferulic acid	194.18	4	2	1.36	0	0.85
Methylferulic acid	208.21	4	1	1.83	0	0.85
Oxooctadecadienoic acid	294.43	3	1	4.75	0	0.85
13-Oxooctadecadienoic acid	294.43	3	1	4.58	0	0.85
(6E,8Z)-5-oxooctadecadienoic acid	294.43	3	1	4.58	0	0.85

LogP: water–octanol partition coefficient.

**Table 4:** Molecular docking simulation results.

Compounds	∆G Energy (kcal/mol)			
	5-HT_1A_	5-HT_2A_	5-HT_3_	D_2_R
4-Hydroxybenzoic acid	−5.6	−5.9	−6.3	−5.8
3-Hydroxybenzoic acid	−5.5	−5.8	−6.4	−5.5
Coumaric acid	−6.4	−6.0	−7.1	−6.4
Ferulic acid	−6.4	−6.3	−7.0	−6.5
Methylferulic acid	−6.1	−6.0	−6.4	−5.6
Oxooctadecadienoic acid	−6.1	−5.9	−6.0	−5.0
13-Oxooctadecadienoic acid	−6.7	−5.1	−6.4	−4.6
(6E,8Z)-5-oxooctadecadienoic acid	−6.4	−4.8	−6.7	−5.3
Standard inhibitors	−9.5 ^(a)^	−8.4 ^(b)^	−9.0 ^(c)^	−7.6 ^(d)^
Redocking RMSD values (Å)	0.79	0.76	0.88	0.71

^(a)^ Way100635, ^(b)^ ketanserin, ^(c)^ ondansetron, ^(d)^ haloperidol.

## Data Availability

All data generated and analyzed are included within this research article.

## Supplementary Materials

The following supporting information can be downloaded at: https://www.mdpi.com/article/10.3390/ph19020329/s1. Figure S1. LC-MS Extracted Ion Chromatograms (EIC) of the detected compounds from *A. gratissima* active fractions. Figure S2. Characteristic UV spectra of the major compounds tentatively identified in *A. gratissima* var. *gratissima*. Figure S3. Results obtained in the redocking analysis. (a) 5-HT_1A_ (PDB: 7E2Y). (b) 5HT_2A_ (PDB: 8UWL). (c) 5-HT_3_ (PDB: 6Y1Z). (d) D_2_R (PDB: 8IRS). Figure S4. Tridimensional representation of complexes with the highest binding affinity and standard inhibitors. (a) 5-HT_1A_ receptor binding to 13-Oxooctadecadienoic acid (green), Way100635 (yellow). (b) 5-HT_2A_ receptor binding to Ferulic acid (red), Ketanserin (yellow). (c) 5-HT_3_ receptor binding to Coumaric acid (orange), Ondansetron (yellow). (d) D_2_R receptor binding to Ferulic acid (blue), Haloperidol (yellow). Figure S5. Druggable site identified in receptors. (a) 5-HT1A receptor. (b) 5-HT2A receptor. (c) 5-HT3 receptor. (d) D2R receptor. Table S1. Compounds selected for in silico analysis. Table S2. Values of the dissociation constant (Kdcalc) calculated from the ∆G values. Table S3. Druggable site properties identified in receptors.

## Abbreviations

The following abbreviations are used in this manuscript:

Agg	*Aloysia gratissima* var. *gratissima*
FST	Forced Swimming Test
TST	Tail Suspension Test
UPLC	Ultra-Performance Liquid Chromatography
MS	Mass Spectrum
AggHex	Hexane Fraction
AggDCM	Dichloromethane Fraction
AggAcE	Ethyl Acetate Fraction
AggBu	Butanol Fraction
ADME	Absorption, Distribution, Metabolism, and Excretion
GI-A	Gastrointestinal Absorption
BBB-P	Blood–Brain Barrier Permeant
Pgp-S	P-glycoprotein Substrate
CYP450	Cytochrome P450

## References

[1] Depressive Disorder (Depression). https://www.who.int/news-room/fact-sheets/detail/depression.

[2] Evans-Lacko S., Aguilar-Gaxiola S., Al-Hamzawi A., Alonso J., Benjet C., Bruffaerts R., Chiu W.T., Florescu S., de Girolamo G., Gureje O. (2018). Socio-economic variations in the mental health treatment gap for people with anxiety, mood, and substance use disorders: Results from the WHO World Mental Health (WMH) surveys. Psychol. Med..

[3] Del Pino-Sedeño T., González-Pacheco H., González de León B., Serrano-Pérez P., Acosta Artiles F.J., Valcarcel-Nazco C., Hurtado-Navarro I., Rodríguez Álvarez C., Trujillo-Martín M.M., MAPDep Team (2024). Effectiveness of interventions to improve adherence to antidepressant medication in patients with depressive disorders: A cluster randomized controlled trial. Front. Public Health.

[4] Serretti A. (2024). A critical view on new and future antidepressants. Clin. Psychopharmacol. Neurosci..

[5] Kovich H., Kim W., Quaste A.M. (2023). Pharmacologic Treatment of Depression. Am. Fam. Physician.

[6] Fazilat S., Tahmasbi F., Mirzaei M.R., Sanaie S., Yousefi Z., Asnaashari S., Yaqoubi S., Banagozar Mohammadi A., Araj-Khodaei M. (2024). A systematic review on the use of phytotherapy in managing clinical depression. Bioimpacts.

[7] Dobrek L., Głowacka K. (2023). Depression and its phytopharmacotherapy—A narrative review. Int. J. Mol. Sci..

[8] Remali J., Aizat W.M. (2024). Medicinal plants and plant-based traditional medicine: Alternative treatments for depression and their potential mechanisms of action. Heliyon.

[9] Almohammed O.A., Alsalem A.A., Almangour A.A., Alotaibi L.H., Al Yami M.S., Lai L. (2022). Antidepressants and health-related quality of life (HRQoL) for patients with depression: Analysis of the medical expenditure panel survey from the United States. PLoS ONE.

[10] Zhang Y., Li L., Zhang J. (2020). Curcumin in antidepressant treatments: An overview of potential mechanisms, pre-clinical/clinical trials and ongoing challenges. Basic Clin. Pharmacol. Toxicol..

[11] Lotter J., Möller M., Dean O., Berk M., Harvey B.H. (2020). Studies on haloperidol and adjunctive α-mangostin or raw *Garcinia mangostana* Linn pericarp on biobehavioral markers in an immune-inflammatory model of schizophrenia in male rats. Front. Psychiatry.

[12] Zimath P.L., Dalmagro A.P., Ribeiro T.C.M., da Silva R.M.L., Lopes de Almeida G.R., Malheiros A., da Silva L.M., de Souza M.M. (2020). Antidepressant-like effect of hydroalcoholic extract from barks of *Rapanea ferruginea*: Role of monoaminergic system and effect of its isolated compounds myrsinoic acid A and B. Behav. Brain Res..

[13] Taboada T., Alvarenga N.L., Galeano A.K., Arrúa W.J., Campuzano-Bublitz M.A., Kennedy M.L. (2022). In Vivo antidepressant-like effect assessment of two *Aloysia* species in mice and LCMS chemical characterization of ethanol extract. Molecules.

[14] Farahani M.S., Bahramsoltani R., Farzaei M.H., Abdollahi M., Rahimi R. (2015). Plant-derived natural medicines for the management of depression: An overview of mechanisms of action. Rev. Neurosci..

[15] Mouawad M., Nabipur L., Agrawal D.K. (2025). Impact of antidepressants on weight gain: Underlying mechanisms and mitigation strategies. Arch. Clin. Biomed. Res..

[16] Calvi A., Fischetti I., Verzicco I., Belvederi Murri M., Zanetidou S., Volpi R., Coghi P., Tedeschi S., Amore M., Cabassi A. (2021). Antidepressant drugs effects on blood pressure. Front. Cardiovasc. Med..

[17] Njenga C., Ramanuj P.P., de Magalhães F.J.C., Pincus H.A. (2024). New and emerging treatments for major depressive disorder. BMJ.

[18] Costa A.P.R., Vieira C., Bohner L.O.L., Silva C.F., Santos E.C.d.S., De Lima T.C.M., Oliveira C.L. (2013). A proposal for refining the forced swim test in Swiss mice. Prog. Neuro-Psychopharmacol. Biol. Psychiatry.

[19] Can A., Dao D.T., Terrillion C.E., Piantadosi S.C., Bhat S., Gould T.D. (2012). The tail suspension test. J. Vis. Exp..

[20] Rajčević N., Bukvički D., Dodoš T., Marin P.D. (2022). Interactions between Natural Products—A Review. Metabolites.

[21] Kennedy M.L., Taboada T., Snead E., Diarte E.M.G., Coronel C.M., Arrúa W., Heinechen O., Montalbetti Y., Hellion-Ibarrola M.C., Ibarrola D.A. (2021). Evaluation of methanol extract of Aloysia gratissima var. gratissima leaves on behavior and anxiety in mice. Int. J. Pharm. Sci. Res..

[22] Perez-Caballero L., Torres-Sanchez S., Romero-López-Alberca C., González-Saiz F., Mico J.A., Berrocoso E. (2019). Monoaminergic system and depression. Cell Tissue Res..

[23] Luscher B., Shen Q., Sahir N. (2011). The GABAergic deficit hypothesis of major depressive disorder. Mol. Psychiatry.

[24] Liwinski T., Lang U.E., Brühl A.B., Schneider E. (2023). Exploring the therapeutic potential of gamma-aminobutyric acid in stress and depressive disorders through the gut–brain axis. Biomedicines.

[25] Sharp T., Collins H. (2023). Mechanisms of SSRI therapy and discontinuation. Curr. Top. Behav. Neurosci..

[26] Delva N.C., Stanwood G.D. (2021). Dysregulation of brain dopamine systems in major depressive disorder. Exp. Biol. Med..

[27] Zeni A.L.B., Zomkowski A.D.E., Dal-Cim T., Maraschin M., Rodrigues A.L., Tasca C.I. (2011). Antidepressant-like and neuro-protective effects of *Aloysia gratissima*: Investigation of involvement of l-arginine-nitric oxide-cyclic guanosine mono-phosphate pathway. J. Ethnopharmacol..

[28] Zeni A., Zomkowski A., Maraschin M., Tasca C., Rodrigues A. (2013). Evidence of the involvement of the monoaminergic systems in the antidepressant-like effect of *Aloysia gratissima*. J. Ethnopharmacol..

[29] Moroni P., O’Leary N., Filloy J. (2016). Species delimitation in the Aloysia gratissima complex (Verbenaceae) following the phylogenetic species concept. Bot. J. Linn. Soc..

[30] Gruz J., Novák O., Strnad M. (2008). Rapid analysis of phenolic acids in beverages by UPLC–MS/MS. Food Chem..

[31] Schreiner G.E., Schmitt E.G., Brittes G.E., dos Santos L.S., Maders L.T., Gonçalves I.L., de Moura Sarmento S.M., Dartora N., Manfredini V. (2024). Neurotransmitter availability and anti-inflammatory and antioxidant effects of subacute administration of *Aloysia gratissima* (Gillies & Hook) Tronc. and rutin in female Wistar rats. BioChem.

[32] Abu-Reidah I.M., Arráez-Román D., Warad I., Fernández-Gutiérrez A., Segura-Carretero A. (2017). UHPLC/MS2-based approach for the comprehensive metabolite profiling of bean (*Vicia faba* L.) by-products: A promising source of bioactive constituents. Food Res. Int..

[33] Strack D., Hartfeld F., Austenfeld F.A., Grotjahn L., Wray V. (1985). Coumaroyl-, caffeoyl-and feruloyltartronates and their accumulation in mung bean. Phytochemistry.

[34] Abu-Reidah I.M., Arráez-Román D., Al-Nuri M., Warad I., Segura-Carretero A. (2019). Untargeted metabolite profiling and phytochemical analysis of *Micromeria fruticosa* L. (Lamiaceae) leaves. Food Chem..

[35] Desta K.T., Kim G.S., Abd El-Aty A.M., Raha S., Kim M.B., Jeong J.H., Warda M., Hacımüftüoğlu A., Shin H.C., Shim J.H. (2017). Flavone polyphenols dominate in *Thymus schimperi* Ronniger: LC–ESI–MS/MS characterization and study of anti-proliferative effects of plant extract on AGS and HepG2 cancer cells. J. Chromatogr. B Anal. Technol. Biomed. Life Sci..

[36] Jaensch M., Jakupovic J., Sanchez H., Dominguez X.A. (1990). Diterpenes from *Hoffmannia strigillosa*. Phytochemistry.

[37] Abdel Ghani A.E., Al-Saleem M.S.M., Abdel-Mageed W.M., AbouZeid E.M., Mahmoud M.Y., Abdallah R.H. (2023). UPLC-ESI-MS/MS Profiling and cytotoxic, antioxidant, anti-inflammatory, antidiabetic, and antiobesity activities of the non-polar fractions of *Salvia hispanica* L. aerial parts. Plants.

[38] Snook M., Blum M., Whitman D., Arrendale R., Costello C., Harwood J. (1993). Caffeoyltartronic acid from catnip (*Nepeta cataria*): A precursor for catechol in lubber grasshopper (*Romalea guttata*) defensive secretions. J. Chem. Ecol..

[39] Terencio M., Giner R., Sanz M., Máñez S., Ríos J. (1993). On the occurrence of caffeoyltartronic acid and other phenolics in *Chondrilla juncea*. Z. Naturforsch..

[40] Bahramsoltani R., Rostamiasrabadi P., Shahpiri Z., Marques A., Rahimi R., Farzaei M. (2018). *Aloysia citrodora* Palau (*Lemon verbena*): A review of phytochemistry and pharmacology. J. Ethnopharmacol..

[41] Schuster M.F., Schreiner G.E., Dartora N., Zamin L.L. (2025). Antiglioma activity from *Aloysia virgata* (Ruíz & Pavón) Juss. Pharmacol. Res.-Nat. Prod..

[42] Wasowski C., Marder M. (2011). Central nervous system activities of two diterpenes isolated from *Aloysia virgata*. Phytomedicine.

[43] Feng J., Yang Q., Chen M., Ning L., Wang Y., Luo D., Hu D., Lin Q., He F. (2025). Study of the correlation between the anti–ischemic stroke mechanism of 4-hydroxybenzaldehyde and its response to reactive oxygen species in brain metabolism. J. Pharmacol. Exp. Ther..

[44] Xu C., Feng J., Sun H., Yan M., Yang Q., Zhou X., Yang J., He F., Lin Q. (2024). Pharmacokinetics of 4-Hydroxybenzaldehyde in Normal and Cerebral Ischemia–Reperfusion Injury Rats Based on Microdialysis Technique. Eur. J. Drug Metab. Pharmacokinet..

[45] Le Sayec M., Carregosa D., Khalifa K., de Lucia C., Aarsland D., Santos C.N., Rodriguez-Mateos A. (2023). Identification and quantification of (poly) phenol and methylxanthine metabolites in human cerebrospinal fluid: Evidence of their ability to cross the BBB. Food Funct..

[46] Ghaderi S., Gholipour P., Komaki A., Salehi I., Rashidi K., Khoshnam S.E., Rashno M. (2022). p-Coumaric acid ameliorates cognitive and non-cognitive disturbances in a rat model of Alzheimer’s disease: The role of oxidative stress and inflammation. Int. Immunopharmacol..

[47] Karademir Y., Mackie A., Tuohy K., Dye L. (2024). Effects of Ferulic Acid on cognitive function: A systematic review. Mol. Nutr. Food Res..

[48] Botti G., Catenacci L., Dalpiaz A., Randi L., Bonferoni M.C., Perteghella S., Beggiato S., Ferraro L., Pavan B., Sorrenti M. (2025). Nasal Administration of a Nanoemulsion Based on Methyl Ferulate and Eugenol Encapsulated in Chitosan Oleate: Uptake Studies in the Central Nervous System. Pharmaceutics.

[49] Eccles J.A., Baldwin W.S. (2022). Detoxification cytochrome P450s (CYPs) in families 1–3 produce functional oxylipins from polyunsaturated fatty acids. Cells.

[50] Volkamer A., Kuhn D., Grombacher T., Rippmann F., Rarey M. (2012). Combining global and local measures for structure-based druggability predictions. J. Chem. Inf. Model..

[51] Ayipo Y.O., Alananzeh W.A., Ahmad I., Patel H., Mordi M.N. (2023). Structural modelling and in silico pharmacology of β-carboline alkaloids as potent 5-HT_1A_ receptor antagonists and reuptake inhibitors. J. Biomol. Struct. Dyn..

[52] Gandhimathi A., Sowdhamini R. (2015). Molecular modelling of human 5-hydroxytryptamine receptor (5-HT_2A_) and virtual screening studies towards the identification of agonist and antagonist molecules. J. Biomol. Struct. Dyn..

[53] Bielenica A., Kędzierska E., Koliński M., Kmiecik S., Koliński A., Fiorino F., Severino B., Magli E., Corvino A., Rossi I. (2016). 5-HT_2_ receptor affinity, docking studies and pharmacological evaluation of a series of 1,3-disubstituted thiourea derivatives. Eur. J. Med. Chem..

[54] Basak S., Gicheru Y., Kapoor A., Mayer M.L., Filizola M., Chakrapani S. (2019). Molecular mechanism of setron-mediated inhibition of full-length 5-HT_3_A receptor. Nat. Commun..

[55] Im D., Inoue A., Fujiwara T., Nakane T., Yamanaka Y., Uemura T., Mori C., Shiimura Y., Kimura K.T., Asada H. (2020). Structure of the dopamine D_2_ receptor in complex with the antipsychotic drug spiperone. Nat. Commun..

[56] Portilla-Martínez A., Ortiz-Flores M., Hidalgo I., González-Ruiz C., Ceballos G., Nájera N. (2020). Defining pharmacological terms based on receptor ligand interactions. Cardiovasc. Metab. Sci..

[57] Hashem S., Dougha A., Tufféry P. (2025). Ligand-Induced Biased Activation of GPCRs: Recent Advances and New Directions from In Silico Approaches. Molecules.

[58] Kim Y., Kim S. (2023). Target discovery using deep learning-based molecular docking and predicted protein structures with alphafold for novel antipsychotics. Psychiatry Investig..

[59] Karim N., Khan I., Abdelhalim A., Khan A., Halim S.A. (2018). Antidepressant potential of novel flavonoids derivatives from sweet violet (*Viola odorata* L): Pharmacological, biochemical and computational evidences for possible involvement of serotonergic mechanism. Fitoterapia.

[60] Zhao X., Wang C., Zhang J.F., Liu L., Liu A.M., Ma Q., Zhou W.H., Xu Y. (2014). Chronic curcumin treatment normalizes depression-like behaviors in mice with mononeuropathy: Involvement of supraspinal serotonergic system and GABA_A_ receptor. Psychopharmacology.

[61] Kim S., Thiessen P.A., Bolton E.E., Chen J., Fu G., Gindulyte A., Han L., He J., He S., Shoemaker B.A. (2016). PubChem substance and compound databases. Nucleic Acids Res..

[62] Lipinski C.A. (2004). Lead-and drug-like compounds: The rule-of-five revolution. Drug Discov. Today Technol..

[63] Daina A., Michielin O., Zoete V. (2017). Swiss ADME: A free web tool to evaluate pharmacokinetics, drug-likeness and medicinal chemistry friendliness of small molecules. Sci. Rep..

[64] Fryc M., Kluzik D., Czopek A., Jończyk J., Zagórska A. (2025). Serotonin 2A (5-HT_2A_) receptor as evolving biological target: Function, structure, ligands and role in the therapy of neuropsychiatric diseases. Neuropharmacology.

[65] Mesoy S.M., Lummis S.C. (2023). 5-HT_3_ Receptors. Textbook of Ion Channels Volume II.

[66] Volkamer A., Griewel A., Grombacher T., Rarey M. (2010). Analyzing the topology of active sites: On the prediction of pockets and subpockets. J. Chem. Inf. Model..

[67] Hanwell M.D., Curtis D.E., Lonie D.C., Vandermeersch T., Zurek E., Hutchison G.R. (2012). Avogadro: An advanced semantic chemical editor, visualization, and analysis platform. J. Cheminformatics.

[68] Trott O., Olson A.J. (2010). AutoDock Vina: Improving the speed and accuracy of docking with a new scoring function, efficient optimization, and multithreading. J. Comput. Chem..

[69] Choudhury A., Das N.C., Patra R., Bhattacharya M., Ghosh P., Patra B.C., Mukherjee S. (2021). Exploring the binding efficacy of ivermectin against the key proteins of SARS-CoV-2 pathogenesis: An in silico approach. Future Virol..

[70] National Research Councill (2011). Guide for the Care and Use of Laboratory Animals.

