# Supplementary material for: Involvement of Serotonergic and Dopaminergic Systems in Aloysia gratissima var. gratissima: Antidepressant-like Effect, UPLC-DAD-MS Chemical Characterization, and Computational Evidence
Source: Pharmaceuticals (Basel). 2026 Feb 17;19(2):329. doi: 10.3390/ph19020329 (PMC12943621; doi:10.3390/ph19020329)
Supplement: Supplementary file 1 [file pharmaceuticals-19-00329-s001.zip › pharmaceuticals-4096447-supplementary.pdf]

pharmaceuticals

MDPI

Article

Involvement of serotonergic and dopaminergic systems in *Aloysia gratissima* var. *gratissima* antidepressant-like effect, UPLC-DAD-MS chemical characterization and computational evidence.

Miguel A. Campuzano-Bublitz[1≠], Alberto Burgos-Edwards[2≠], Elvio Gayozo[3≠], Adelian A. Acosta[1], Rodrigo S. Paredes[1], Alex D. Campuzano-Kennedy[1], Antonia K. Galeano[1], Yenny P. González[4], Nelson Alvarenga[2], Teresa Taboada-Jara[1], and María L. Kennedy[1,*]

Figure S1. LC-MS Extracted Ion Chromatograms (EIC) of the detected compounds from *A. gratissima* active fractions.

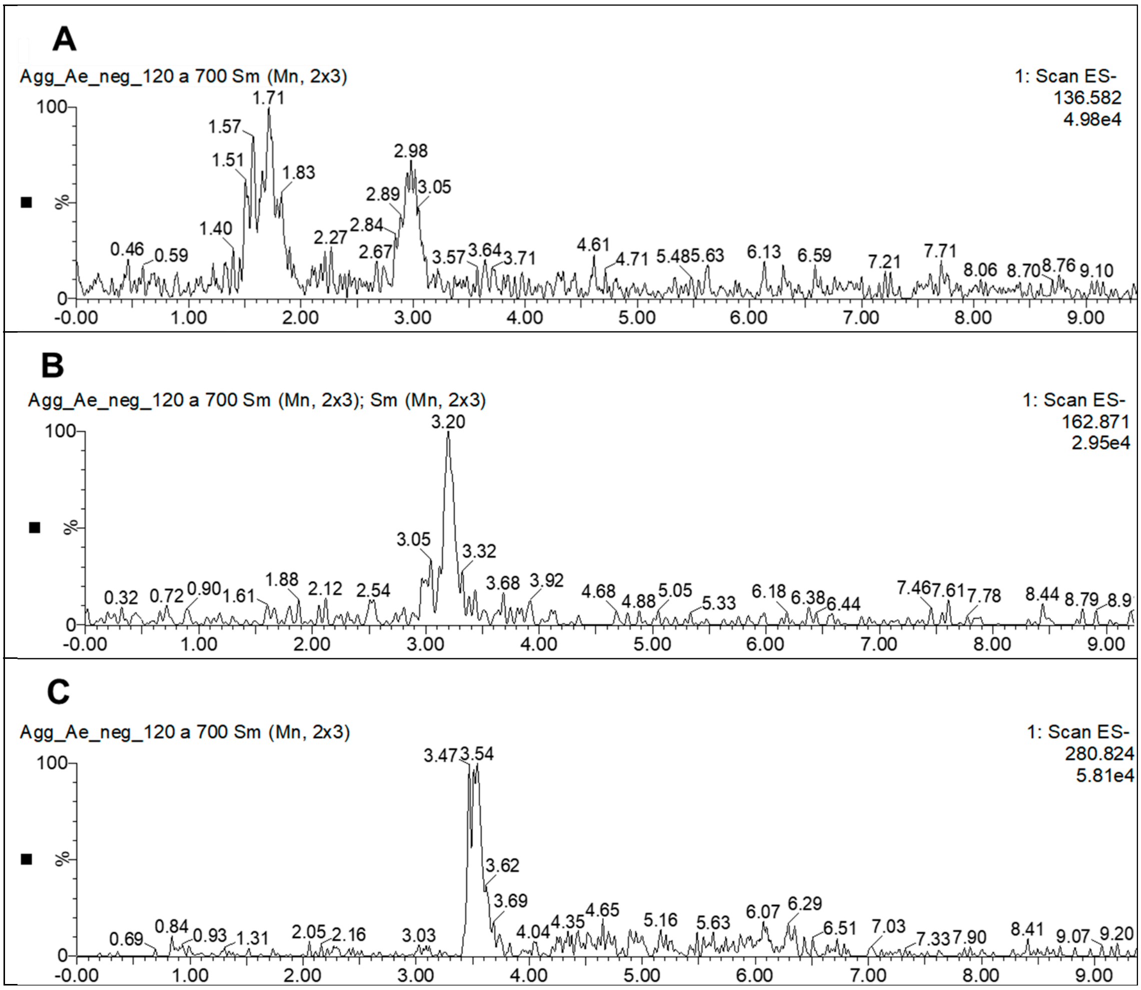

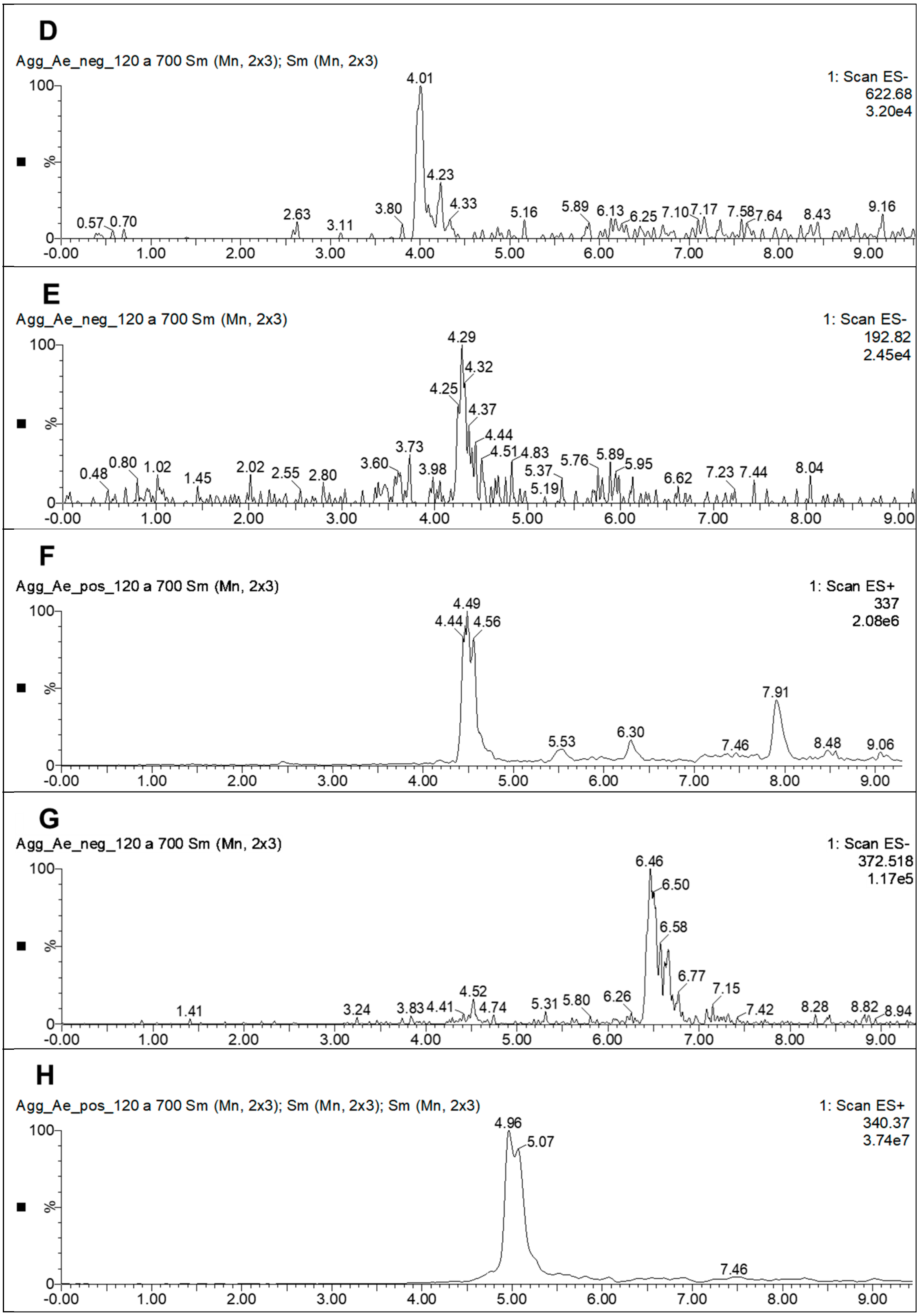
D
Agg_Ae_neg_120 a 700 Sm (Mn, 2x3); Sm (Mn, 2x3)
1: Scan ES-
622.68
3.20e4
E
Agg_Ae_neg_120 a 700 Sm (Mn, 2x3)
1: Scan ES-
192.82
2.45e4
F
Agg_Ae_pos_120 a 700 Sm (Mn, 2x3)
1: Scan ES+
337
2.08e6
G
Agg_Ae_neg_120 a 700 Sm (Mn, 2x3)
1: Scan ES-
372.518
1.17e5
H
Agg_Ae_pos_120 a 700 Sm (Mn, 2x3); Sm (Mn, 2x3); Sm (Mn, 2x3)
1: Scan ES+
340.37
3.74e7

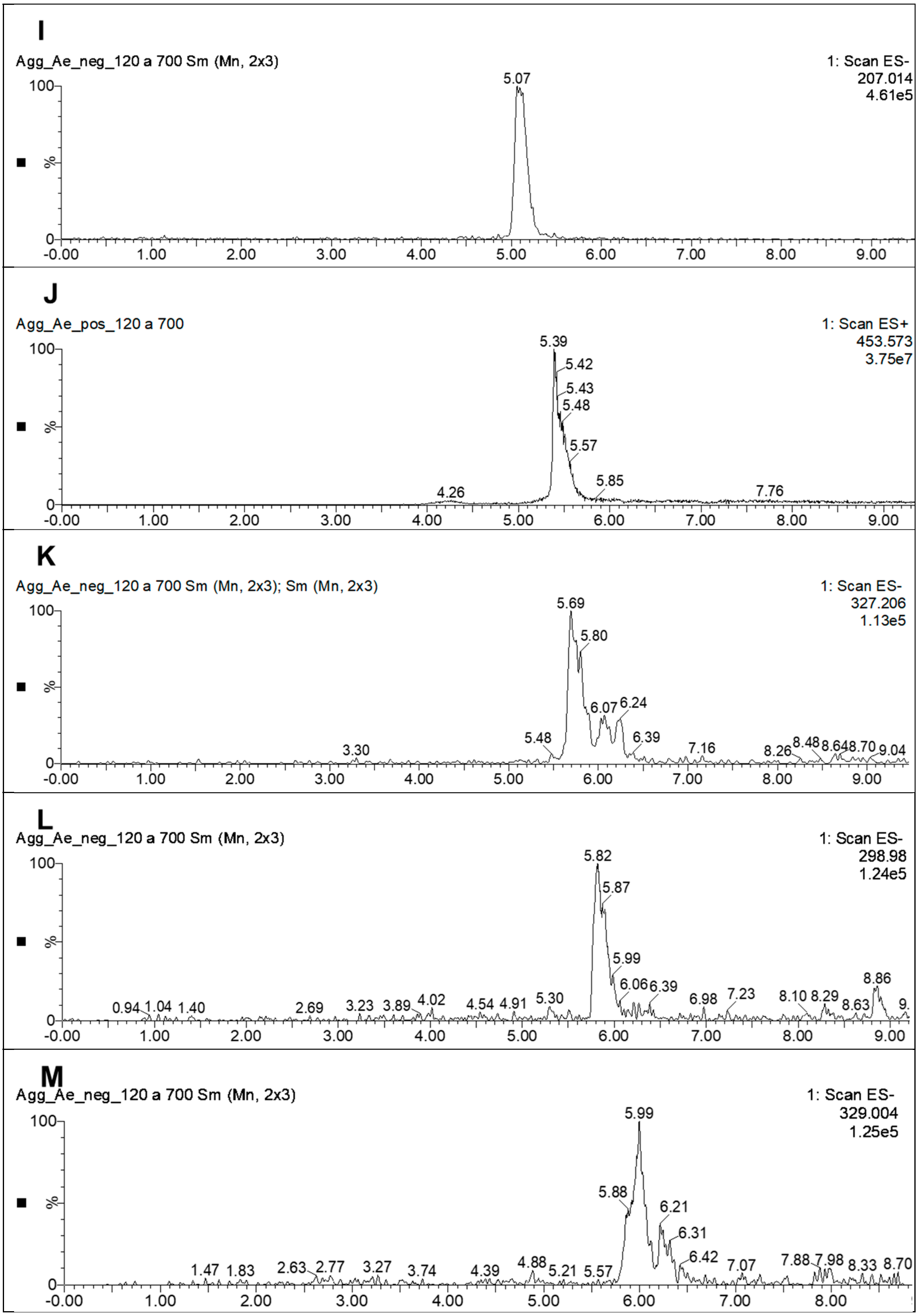
I
Agg_Ae_neg_120 a 700 Sm (Mn, 2x3)
1: Scan ES-
207.014
4.61e5
100
%
0
5.07
-0.00 1.00 2.00 3.00 4.00 5.00 6.00 7.00 8.00 9.00
J
Agg_Ae_pos_120 a 700
1: Scan ES+
453.573
3.75e7
100
%
0
5.39 5.42 5.43 5.48 5.57 5.85 4.26 7.76
-0.00 1.00 2.00 3.00 4.00 5.00 6.00 7.00 8.00 9.00
K
Agg_Ae_neg_120 a 700 Sm (Mn, 2x3); Sm (Mn, 2x3)
1: Scan ES-
327.206
1.13e5
100
%
0
5.69 5.80 6.07 6.24 5.48 6.39 3.30 7.16 8.26 8.48 8.64 8.70 9.04
-0.00 1.00 2.00 3.00 4.00 5.00 6.00 7.00 8.00 9.00
L
Agg_Ae_neg_120 a 700 Sm (Mn, 2x3)
1: Scan ES-
298.98
1.24e5
100
%
0
5.82 5.87 5.99 6.06 6.39 0.94 1.04 1.40 2.69 3.23 3.89 4.02 4.54 4.91 5.30 6.98 7.23 8.10 8.29 8.63 8.86 9.
-0.00 1.00 2.00 3.00 4.00 5.00 6.00 7.00 8.00 9.00
M
Agg_Ae_neg_120 a 700 Sm (Mn, 2x3)
1: Scan ES-
329.004
1.25e5
100
%
0
5.99 5.88 6.21 6.31 6.42 1.47 1.83 2.63 2.77 3.27 3.74 4.39 4.88 5.21 5.57 7.07 7.88 7.98 8.33 8.70
-0.00 1.00 2.00 3.00 4.00 5.00 6.00 7.00 8.00

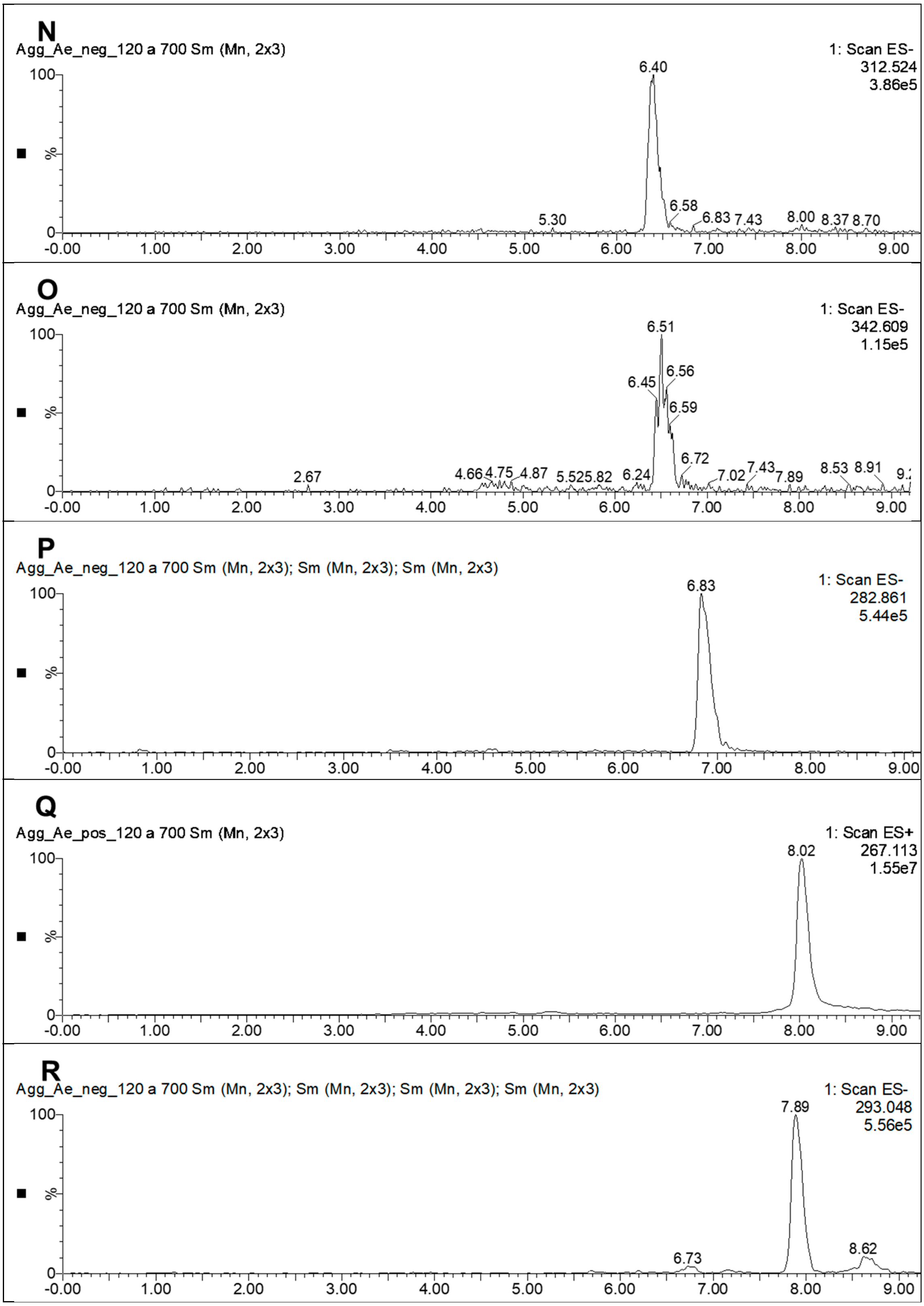
N
Agg_Ae_neg_120 a 700 Sm (Mn, 2x3)
1: Scan ES-
312.524
3.86e5
100
%
0
6.40
5.30
6.58
6.83
7.43
8.00
8.37
8.70
-0.00
1.00
2.00
3.00
4.00
5.00
6.00
7.00
8.00
9.00
O
Agg_Ae_neg_120 a 700 Sm (Mn, 2x3)
1: Scan ES-
342.609
1.15e5
6.51
6.45
6.56
6.59
6.72
2.67
4.66
4.75
4.87
5.52
5.82
6.24
7.02
7.43
7.89
8.53
8.91
P
Agg_Ae_neg_120 a 700 Sm (Mn, 2x3); Sm (Mn, 2x3); Sm (Mn, 2x3)
1: Scan ES-
282.861
5.44e5
6.83
Q
Agg_Ae_pos_120 a 700 Sm (Mn, 2x3)
1: Scan ES+
267.113
1.55e7
8.02
R
Agg_Ae_neg_120 a 700 Sm (Mn, 2x3); Sm (Mn, 2x3); Sm (Mn, 2x3); Sm (Mn, 2x3)
1: Scan ES-
293.048
5.56e5
7.89
6.73
8.62

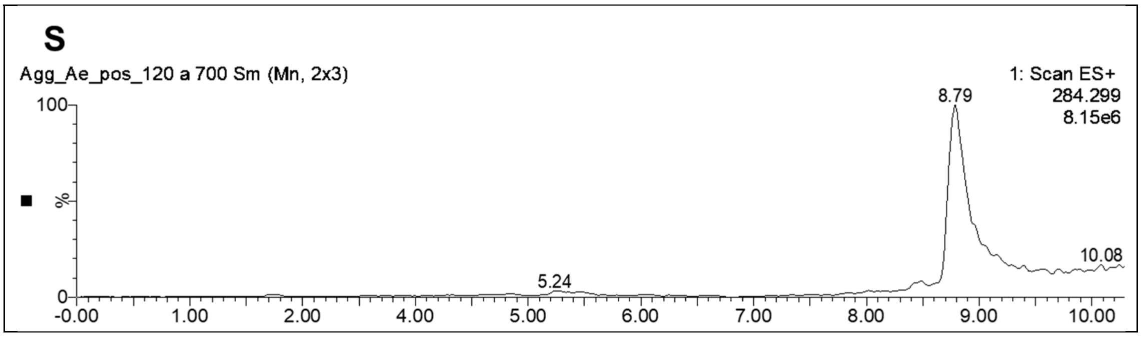

#Compounds: (A) Hydroxybenzoic acid and isomer; (B) Coumaric acid; (C) caffeoyl tartronic acid; (D) Verbascoside; (E) Ferulic acid; (F) Hydroxymethylhoffmanniaketone and isomer; (G) Methylsudachitin; (H) Unknown; (I) Methyl ferulic acid; (J) Unknown; (K) Coumaroyl derivative; (L) Methoxyapigenin; (M) Hydroxy-di-O-methylluteolin; (N) Dihydroxy-dimethoxyflavone; (O) Dihydroxy-trimethoxy flavone; (P) Methyl apigenin; (Q) Unknown; (R) Oxooctadeca-dienoic acid; (S) Unknown.

Figure S2. Characteristic UV spectra of the major compounds tentatively identified in *A. gratissima* var. *gratissima*.

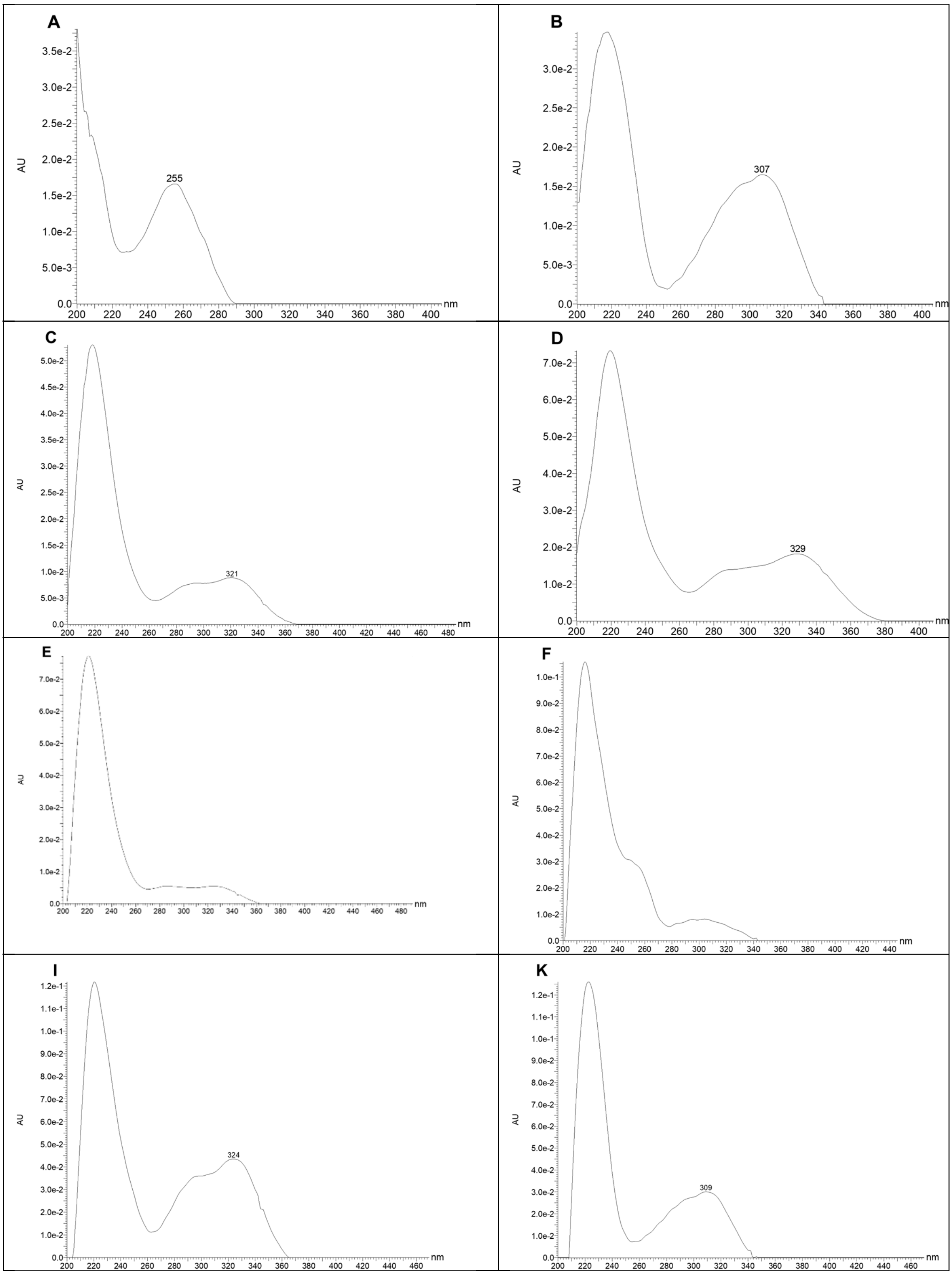

pharmaceuticals

MDPI

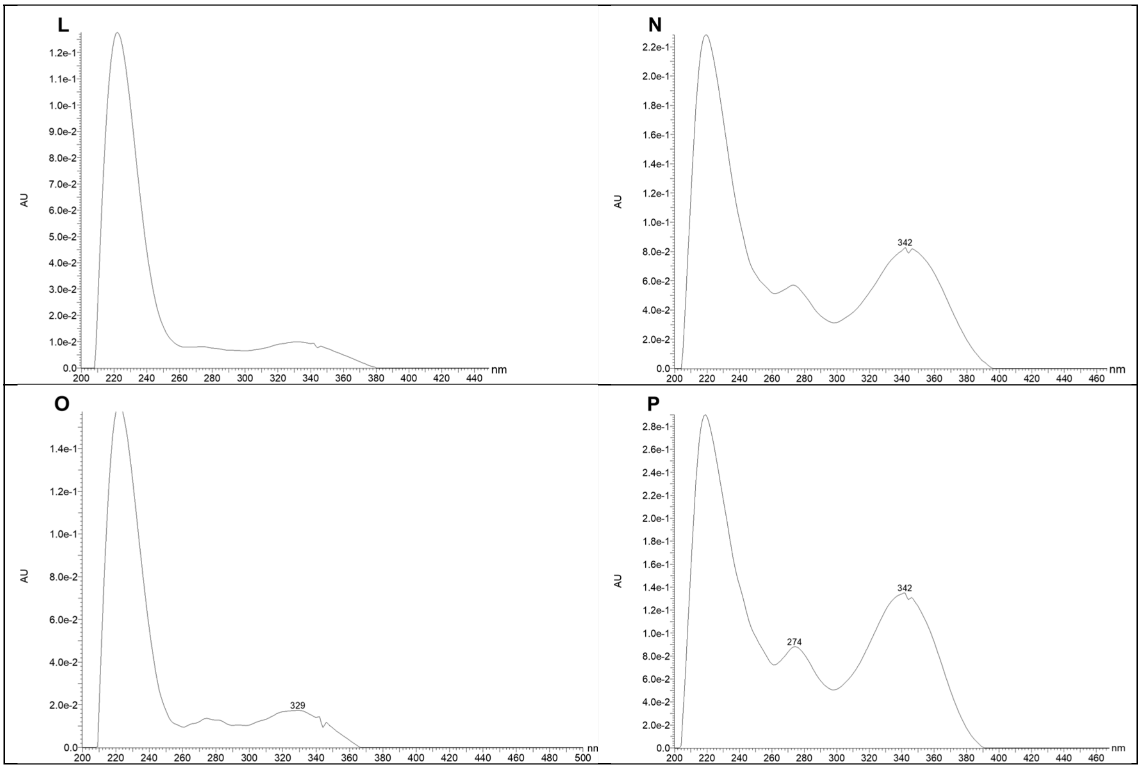

#Compounds: (A) Hydroxybenzoic acid and isomer; (B) Coumaric acid; (C) caffeoyl tartronic acid; (D) Verbascoside; (E) Ferulic acid; (F) Hydroxymethylhoffmanniaketone; (I) Methyl ferulic acid; (K) Coumaroyl derivative; (L) Methoxyapigenin; (N) Dihydroxy-dimethoxyflavone; (O) Dihydroxy-trimethoxy flavone; (P) Methyl apigenin.

pharmaceuticals

Figure S3. Results obtained in the redocking analysis. (a) 5-HT_{1A} (PDB: 7E2Y). (b) $5HT_{2A}$ (PDB: 8UWL). (c) 5-HT_3 (PDB: 6Y1Z). (d) D_2R (PDB: 8IRS).

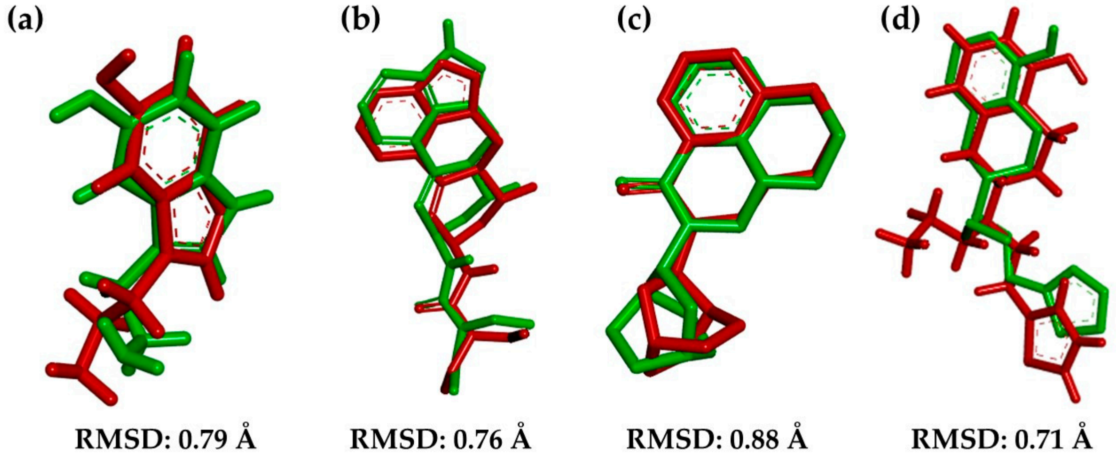

pharmaceuticals

Figure S4. Tridimensional representation of complexes with the highest binding affinity and standard inhibitors. (a) 5-HT_{1A} receptor binding to 13-Oxooctadecadienoic acid (green), Way100635 (yellow). (b) 5-HT_{2A} receptor binding to Ferulic acid (red), Ketanserin (yellow). (c) 5-HT_3 receptor binding to Coumaric acid (orange), Ondansetron (yellow). (d) D_2R receptor binding to Ferulic acid (blue), Haloperidol (yellow).

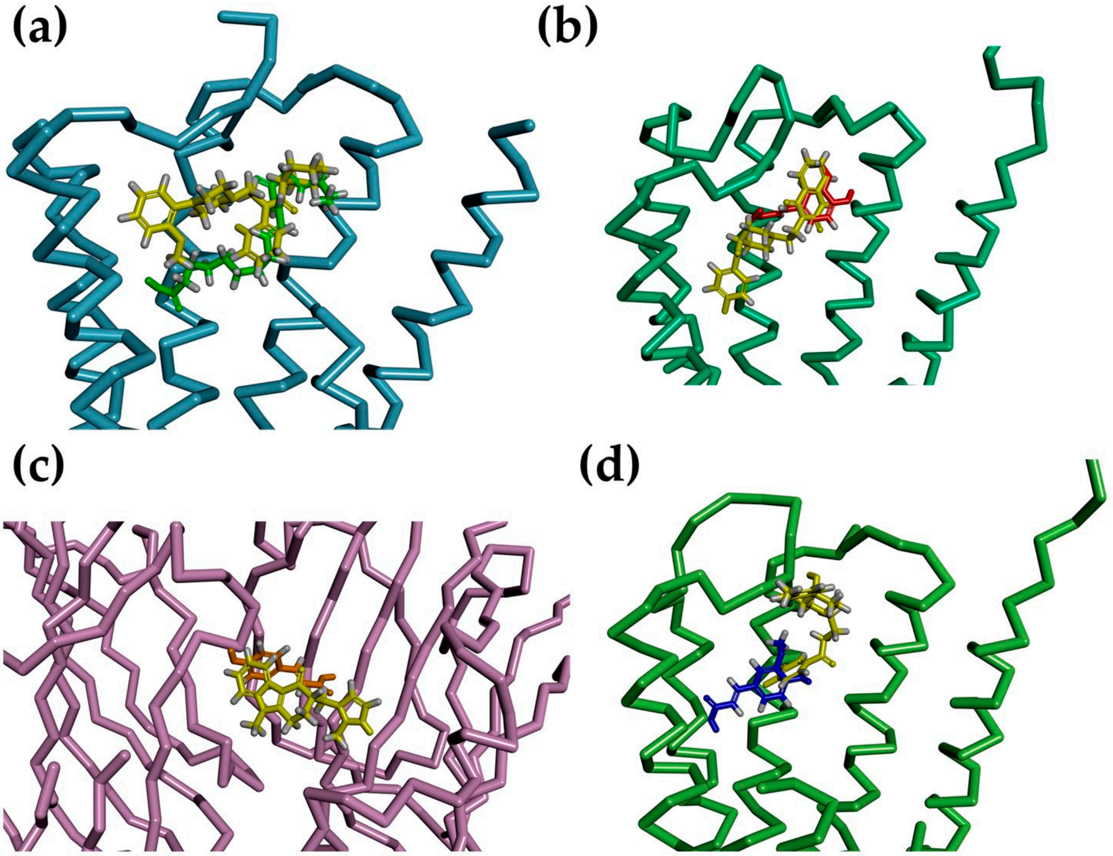

pharmaceuticals

Figure S5. Druggable site identified in receptors. (**a**) 5-HT_{1A} receptor. (**b**) 5-HT_{2A} receptor. (**c**) 5-HT_3 receptor. (**d**) D_2R receptor.

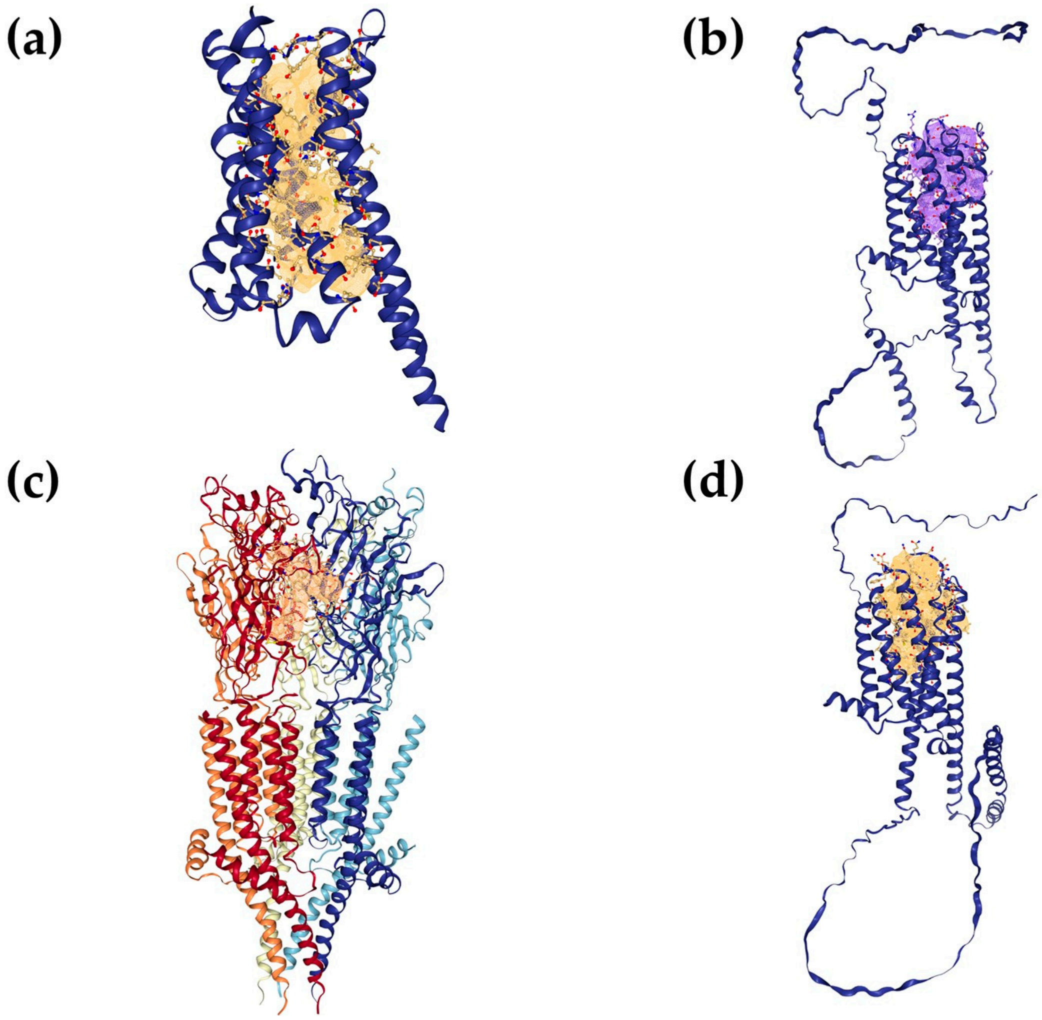

pharmaceuticals

MDPI

Table S1. Compounds selected for *in silico* analysis.

Compounds*	PubChem Compounds†	PubChem CID
Hydroxybenzoic acid	4-Hydroxybenzoic Acid	135
Hydroxybenzoic acid 2	3-Hydroxybenzoic Acid	7420
Coumaric acid	Coumaric acid	637542
2-O-caffeoyl-hydroxymalonic acid (caffeoyl tartronic acid)	2-O-caffeoyl-hydroxymalonic acid (caffeoyl tartronic acid)	131752962
Verbascoside	Verbascoside	5281800
Ferulic acid	Ferulic acid	445858
Hydroxymethylhoffmanniaketone	Hydroxymethylhoffmanniaketone	162954573
Methylsudachitin	Methylsudachitin	181092
Methylferulic acid	Methylferulic acid	717531
Methoxyapigenin[a]	3'-Methoxyapigenin	5280666
	6-Methoxyapigenin	5281628
	3-Methoxyapigenin	5280862
	8-Methoxyapigenin	5322078
Hydroxy-di-O-methylluteolin	Hydroxy-di-O-methylluteolin	5320945
Dihydroxy-dimethoxyflavone	Dihydroxy-dimethoxyflavone	123885531
Dihydroxy-trimethoxy flavone	Dihydroxy-trimethoxyflavone	91248359
Methyl apigenin	Methylapigenin	15661821
Hydroxymethylhoffmanniaketone isomer	Hydroxymethylhoffmanniaketone isomer	101394717
Oxooctadecadienoic acid[b]	Oxooctadecadienoic acid	71407514
	13-Oxooctadecadienoic acid	160205
	(6E,8Z)-5-oxooctadecadienoic acid	71751402

*Identified in this study by UPLC-ESI-MS/MS, †Compounds selected for *in silico* analysis, [a]Methoxyapigenin isomers detected in database, [b] Oxooctadeca-dienoic acid isomers detected in database.

pharmaceuticals

Table S2. Values of the dissociation constant (**Kd**$_{calc}$) calculated from the ΔG values.

Compounds	Kd$_{calc}$ (μM)			
	**5-HT$_{1a}$**	**5-HT$_{2a}$**	**5-HT$_{3}$**	**D$_{2}$R**
4-Hydroxybenzoic acid	112.74	69.28	36.19	81.49
3-Hydroxybenzoic acid	132.61	81.49	30.77	132.61
Coumaric acid	30.77	58.89	9.88	30.77
Ferulic acid	30.77	36.19	11.62	26.16
Methylferulic acid	50.07	58.89	30.77	112.74
Oxooctadecadienoic acid	50.07	69.28	58.89	298.59
13-Oxooctadecadienoic acid	18.91	253.85	30.77	571.57
(6E,8Z)-5-oxooctadecadienoic acid	30.77	413.12	18.91	183.48
Standard inhibitors	0.20[(a)]	1.19[(b)]	0.45[(c)]	4.39[(d)]

[(a)]Way100635, [(b)]Ketanserin, [(c)]Ondansetron, [(d)]Haloperidol.

pharmaceuticals

MDPI

Table S3. Druggable site properties identified in receptors.

Receptor	Volume (Å³)	Surface (Å²)	Depth (Å)	Druggable score	Residues
5-HT_{1A}	2244.85	2391.51	26.69	0.82	Ala71, Leu74, Ile75, Ser77, Leu78, Asp82, Val85, Leu90, Ala93, Ala94, Tyr96, Gln97, Phe112, Ile113, Asp116, Val117, Cys119, Cys120, Thr121, Ser123, Ile124, Trp125, His126, Leu127, Cys128, Ala129, Ile130, Ala131, Leu132, Asp133, Arg134, Tyr135, Ile138, Arg148, Ala153, Leu156, Ile157, Leu159, Thr160, Ile163, Ile167, Cys187, Thr188, Ile189, Ser190, Lys191, Tyr195, Thr196, Ser199, Thr200, Ala203, Pro207, Leu208, Leu210, Met211, Leu214, Tyr215, Ile218, Thr343, Lys345, Thr346, Leu347, Ile349, Ile350, Met351, Thr353, Phe354, Leu356, Cys357, Trp358, Leu359, Pro360, Phe361, Phe362, Val364, Ala365, Leu368, Pro369, Met377, Gly382, Ala383, Ile385, Asn386, Trp387, Leu388, Gly389, Tyr390, Asn392, Ser393, Leu395, Asn396, Ile399, Tyr400, Ala401, Phe403, Asn404
5-HT_{2A}	2063.11	1954.87	34.5	0.99	Leu116, Ala119, Asp120, Leu123, Met128, Val130, Ser131, Thr134, Ile135, Tyr139, Arg140, Trp141, Trp151, Ile152, Asp155, Val156, Phe158, Ser159, Thr160, Ser162, Ile163, Leu166, Gly205, Ile206, Ser207, Met208, Pro209, Ile210, Lys223, Glu224, Ser226, Cys227, Leu228, Leu229, Ala230, Asp231, Asp232, Phe234, Val235, Ile237, Gly238, Ser239, Val241, Ala242, Phe243, Ile327, Val328, Leu331, Phe332, Met335, Trp336, Phe339, Phe340, Asn343, Ala346, Val347, Cys349, Lys350, Cys353, Asn354, Glu355, Asn356, Ile358, Gly359, Leu362, Asn363, Val366, Trp367, Gly369, Tyr370, Leu371, Ser372, Ser373, Ala374, Val375, Asn376, Val379
5-HT_3	937.34	959.03	23.78	0.82	Leu58(chain A), Thr59(A), Thr60(A), Asn101(A), Glu102(A), Leu131(A), Val132(A), Thr133(A), Ala134(A), Cys135(A), Ser136(A), Cys149(A), Ser150(A), Leu151(A), Thr152(A), Thr154(A), Ser155(A), Trp156(A), Leu157(A), His158(A), Thr159(A), Ile160(A), Ile163(A), Asn164(A), Ile165(A), Phe199(A), Ile201(A), Ser206(A), Tyr207(A), Ala208(A), Glu209(A), Lys211(A), Val214(A), Ile44(E), Tyr46(chain E), Trp63(E), Tyr64(E), Arg65(E), Tyr126(E), Lys127(E), Arg169(E), Asp177(E), Ser179(E), Ile180(E), Ile182(E)

pharmaceuticals MDPI

D_2R	2224.56	2422.61	39.29	0.81	Tyr37, Leu41, Ile48, Leu76, Ala79, Asp80, Val83, Val87, Trp90, Val91, Leu94, Glu95, Gly98, Glu99, Trp100, Lys101, Phe102, Ser103, Arg104, Cys107, Asp108, Phe110, Val111, Thr112, Asp114, Val115, Met117, Cys118, Thr119, Ser121, Ile122, Leu125, Pro169, Leu170, Gly173, Leu174, Asn175, Asn176, Thr177, Asp178, Gln179, Asn180, Glu181, Cys182, Ile183, Ile184, Ala185, Asn186, Phe189, Val190, Val191, Ser193, Ser194, Ile195, Ser197, Phe198, Tyr199, Val379, Val382, Phe383, Cys386, Trp387, Pro389, Phe390, Phe391, Ile392, HIS394, Ile395, Leu396, Val407, Tyr409, Ser410, Ala411, Phe412, Thr413, Trp414, Leu415, Gly416, Tyr417, Val418, Asn419, Ser420, Val422, Asn423, Ile426
